# MYC regulates CSF1 expression via microRNA 17/20a to modulate tumor-associated macrophages in osteosarcoma

**DOI:** 10.1172/jci.insight.164947

**Published:** 2023-07-10

**Authors:** Bikesh K. Nirala, Tajhal D. Patel, Lyazat Kurenbekova, Ryan Shuck, Atreyi Dasgupta, Nino Rainusso, Cristian Coarfa, Jason T. Yustein

**Affiliations:** 1 Texas Children’s Cancer and Hematology Centers and The Faris D. Virani Ewing Sarcoma Center,; 2 Department of Molecular & Human Genetics, and; 3 Department of Molecular and Cellular Biology, Baylor College of Medicine, Houston, Texas, USA.; 4 Aflac Cancer and Blood Disorders Center of Children’s Healthcare of Atlanta, Emory University, Atlanta, Georgia, USA.

**Keywords:** Oncology, Bone disease, Mouse models, Oncogenes

## Abstract

Osteosarcoma (OS) is the most common primary bone tumor of childhood. Approximately 20%–30% of OSs carry amplification of chromosome 8q24, which harbors the oncogene c-*MYC* and correlates with a poor prognosis. To understand the mechanisms that underlie the ability of MYC to alter both the tumor and its surrounding tumor microenvironment (TME), we generated and molecularly characterized an osteoblast-specific Cre-Lox-Stop-Lox-*c-Myc^T58A^ p53*^fl/+^ knockin genetically engineered mouse model (GEMM). Phenotypically, the *Myc*-knockin GEMM had rapid tumor development with a high incidence of metastasis. MYC-dependent gene signatures in our murine model demonstrated significant homology to the human hyperactivated MYC OS. We established that hyperactivation of MYC led to an immune-depleted TME in OS demonstrated by the reduced number of leukocytes, particularly macrophages. MYC hyperactivation led to the downregulation of macrophage colony-stimulating factor 1, through increased microRNA 17/20a expression, causing a reduction of macrophage population in the TME of OS. Furthermore, we developed cell lines from the GEMM tumors, including a degradation tag–MYC model system, which validated our MYC-dependent findings both in vitro and in vivo. Our studies utilized innovative and clinically relevant models to identify a potentially novel molecular mechanism through which MYC regulates the profile and function of the OS immune landscape.

## Graphical abstract

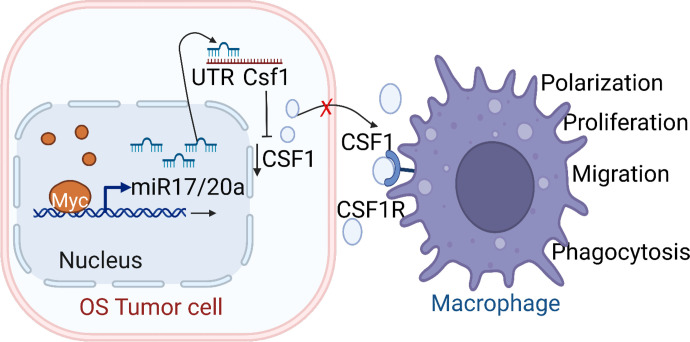

## Introduction

Osteosarcoma (OS) is the most common and highly metastatic primary bone tumor in children and adolescents (1). Despite extensive genomic aberrations, OS has no pathognomonic DNA translocation or targetable mutations (2). Thus, no effective molecularly targeted therapies for OS are currently available. However, many patients with OS present with genetically defined somatic DNA copy number alterations, such as chromosome 8q24 gain, which is noted in about 20% of patients with OS (3, 4). The 8q24 locus harbors the known oncogene *c-MYC* (*MYC*), which directly regulates several protein-coding and noncoding genes important for distinct cellular functions, including cell cycle regulation, protein biogenesis, metabolism, signal transduction, transcription, and translation (5, 6). *MYC* has been found to be deregulated in more than half of human cancers (7). Amplification of the 8q24 region and overexpression of *MYC* is seen in both high-grade premalignancy and invasive tumors and is associated with poor outcome in different human tumor types, including OS (8–12). Besides its effects on intrinsic tumor cell biology, hyperactivation of *MYC* leads to alterations in the tumor immune microenvironment (TME) in multiple cancers (13–15).

Macrophages are abundantly present cells in the TME of solid tumors, including OS, and play multifunctional roles in host defense, tissue repair, apoptosis, and tissue homeostasis by releasing a distinct repertoire of growth factors, cytokines, chemokines, and enzymes (16, 17). In mature adults, macrophages are differentiated from peripheral blood monocytes with the help of a cytokine, macrophage colony-stimulating factor 1 (M-CSF or CSF1). CSF1 not only regulates the differentiation of monocytes to macrophages but also supports monocytes/macrophages’ survival and proliferation, and macrophage motility through interaction with its receptor (CSF1R) (18). The role of intratumor oncogenic MYC in macrophage regulation has been partially explored. MYC has been found to play a key role in alternative macrophage activation (19), but knowledge of the underlying molecular pathways is scarce. Moreover, the role of MYC in the regulation of the macrophage population in the TME of OS is unknown.

We have generated an osteoblast-specific *Myc*-knockin genetically engineered mouse model (GEMM) of OS and molecularly characterized spontaneous OS tumors that arise in this model to identify MYC-dependent intrinsic and extrinsic therapeutic vulnerabilities. The murine molecular profiles were compared with the human tumor transcriptomic profile from the Therapeutically Applicable Research to Generate Effective Treatments (TARGET data set, https://ocg.cancer.gov/programs/target) and R2: Genomics Analysis and Visualization Platform data set (https://hgserver1.amc.nl/cgi-bin/r2/main.cgi?open_page=login) (20). We observed that hyperactivation of MYC was associated with a diminished leukocyte population, particularly the macrophage subpopulation, in the TME of OS. Additionally, we observed the infiltration of the macrophage cells was diminished in the OS TME for both human and mouse tumors. Our results demonstrated the role of cytokine CSF1 in macrophage recruitment to the TME in OS. Subsequently, we identified that MYC-regulated microRNA (miR) 17/20a downregulated CSF1 expression, resulting in the direct downstream effects on intratumor macrophage recruitment. Additionally, MYC was found to regulate macrophage functions, including phagocytosis, through CSF1 regulation. This is the first study to our knowledge that identifies a direct molecular mechanism of macrophage regulation in the OS TME by MYC. In addition, our potentially novel *Myc*-knockin GEMM provides valuable resources to improve our knowledge about the etiology of OS and identification of therapeutic targets for this high-risk subgroup of patients with OS.

## Results

### Development and proteotranscriptomic characterization of a Myc-knockin GEMM of OS.

**Figure 1:**
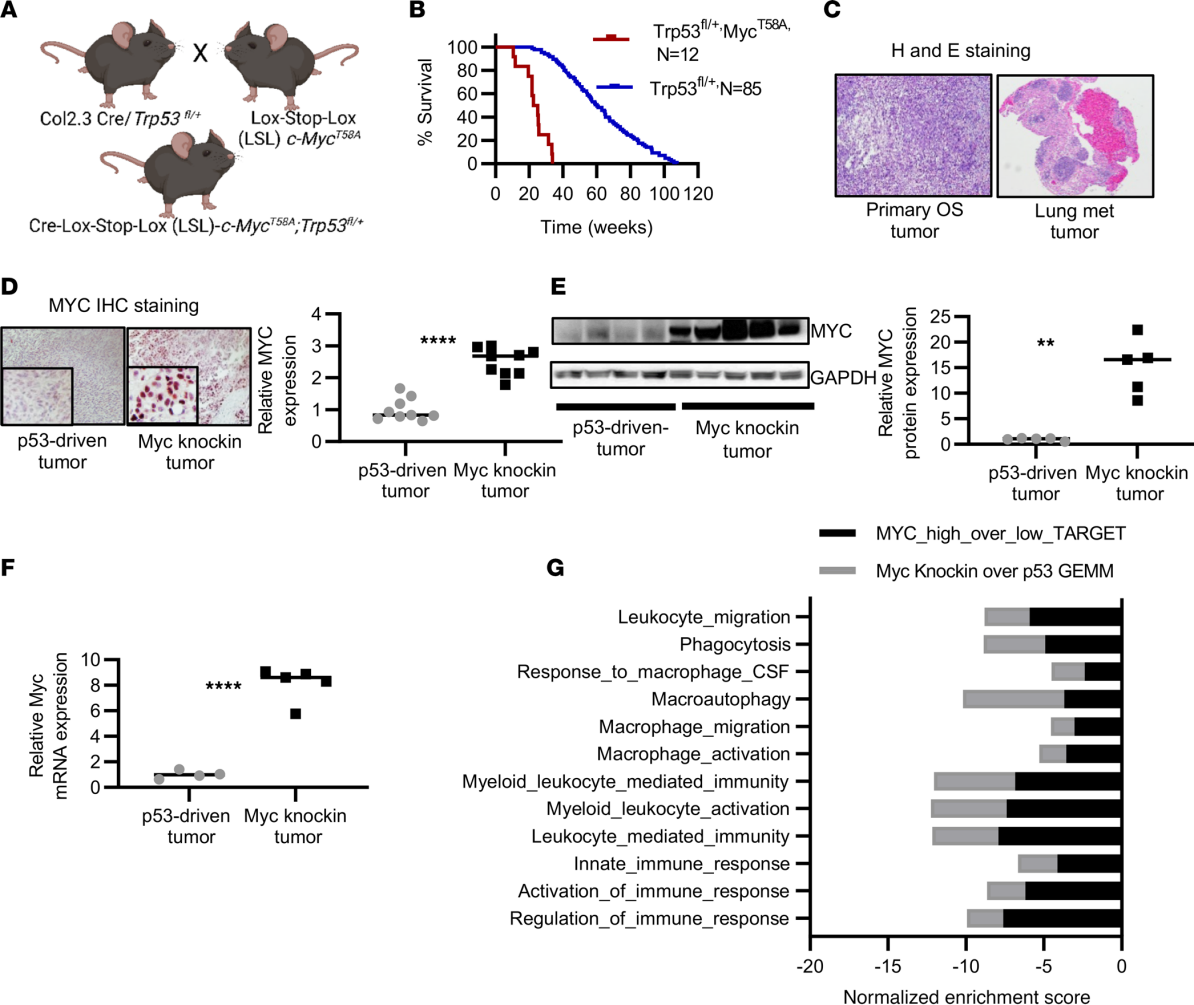
Development and proteotranscriptomic characterization of *Myc*-knockin GEMM of OS. (**A**) Schema of generation of *Myc*-knockin GEMM. (**B**) Kaplan-Meier curve showing accelerated OS development in the *Myc*-knockin (*n* = 12; red) versus heterozygous *Trp53^fl/+^* (*n* = 85; blue) model; log-rank (Mantel-Cox) test was performed for the Kaplan-Meier analyses. (**C**) H&E of the primary tumor (left panel) and associated lung lesions (right panel). Original magnification, 4×. (**D**) IHC staining with MYC in the paraffin-embedded tumor tissue samples showed higher expression in the *Myc*-knockin specimen compared with p53-driven GEMM tumor; quantified expression is shown in the right panel. IHC images were captured at 20× original magnification. Inset image is original magnification, 80×. (**E**) Western blot demonstrating increased MYC protein expression in *Myc*-knockin tumors compared with *Trp53^fl/+^* driven tumor; quantified expression is shown in the right panel. (**F**) Relative mRNA expression analyzed by the RNA sequencing (RNA-Seq) demonstrated increased *Myc* mRNA expression in *Myc*-knockin (*n* = 5) tumors compared with the p53-driven (*n* = 4) tumor sample. (**G**) Gene set enrichment analysis (GSEA) comparison between GEMM tumor tissue samples and the high-*Myc*- versus low-*Myc*-expressing human OS model using the OS TARGET data set. **P < 0.01, ****P < 0.0001.

Previously, we generated a conditional GEMM of OS to understand the molecular pathogenesis of disease development and progression through osteoblast-specific alteration of the *Trp53* gene (21). *Trp53* is a tumor suppressor and estimated to be mutated, or dysregulated, in 80%–90% of OS tumors (22). More recently, efforts have been made to categorize patients with OS into genetically defined subpopulations, including patients with amplified chromosome 8q24.2 region, which harbors the oncogene c-*Myc*. To further understand the role of MYC-dependent molecular and cellular tumor-intrinsic and -extrinsic profiles in OS tumor development and metastasis, we used our prior OS GEMM (referred to as the p53 model in the text). We generated a *Myc*-knockin GEMM by crossing the conditional Col 2.3-Cre *Trp53^fl/+^* mice with Lox-Stop-Lox-*c-Myc^T58A^* mice to generate Cre-Lox-Stop-Lox-*c-Myc^T58A^ Trp53^fl/+^* mice (referred to as *Myc*-knockin GEMM in the text) (Figure 1A).

The *Myc*-knockin GEMM developed rapid-onset OS tumors with a median time to sacrifice of approximately 24 weeks versus 52 weeks for the conditional p53 model (Figure 1B). In addition, we observed a high incidence of pulmonary metastasis (>60%) in the *Myc*-knockin compared with approximately 20% incidence seen in our p53 GEMM. Histological analysis of the primary and metastatic tumors validated the OS histology (Figure 1C). MYC immunohistochemical (IHC) staining showed higher protein expression in the tumor tissue of the *Myc*-knockin specimens as compared with the p53 samples (Figure 1D). Also, a significantly higher level of MYC expression was observed both at the mRNA and at the protein levels in the tumor tissue samples of *Myc*-knockin GEMMs as compared with the p53 GEMM (Figure 1, E and F).

Further, we performed molecular characterization of the GEMMs by analyzing the tumor tissue samples using whole-tumor RNA sequencing (RNA-Seq) and total-proteome analysis. We performed a cross-species transcriptomic comparison of the GEMM molecular signatures with human OS tumors using the OS TARGET data set and R2 Genomic Analysis data set. We noted that 2,743 genes were differentially expressed at the transcriptional level (*P* < 0.05) between *Myc*-knockin and p53 tumors, with 1,055 downregulated and 1,688 upregulated (Supplemental Figure 1A; supplemental material available online with this article; https://doi.org/10.1172/jci.insight.164947DS1). Subsequently, a comparison of gene set enrichment analysis (GSEA) using differentially expressed genes from *Myc*-knockin and p53 GEMM tumors to the OS TARGET data set was performed. This demonstrated our *Myc*-knockin GEMM closely resembled the high-*MYC*-expressing human OS tumor subtype with 3,147 positively and negatively enriched overlapping gene sets between mouse and human samples (Supplemental Figure 1B). Specifically, we identified concurrent alterations in the innate and adaptive immune response, myeloid and leukocyte-mediated immunity, macrophage migration, chemotaxis, differentiation, and CSF signaling for our GEMM and human OS TARGET data set (Figure 1G and Supplemental Table 1). Besides immune-related signatures, which had significantly negative normalized enrichment scores for both our murine and human MYC-hyperactivated data sets, we also observed common enrichment scores in numerous other gene sets/pathways. Besides enrichment of MYC target genes (HALLMARK MYC TARGETS V1 and V2), we found positive enrichment in DNA replication, RNA processing and splicing, and amino acid metabolism, alongside common negative enrichment in cell-cell adhesion, oxidative phosphorylation, fatty acid metabolism, and antigen presentation (Supplemental Table 1). In summary, based on histopathology and proteotranscriptomic analysis, we provide evidence of a strong correlation between the phenotypic and molecular profiles of the murine *Myc*-knockin model and high-*MYC*-expressing patient OS tumors.

### Molecular characterization of OS syngeneic mouse models and cell lines.

**Figure 2:**
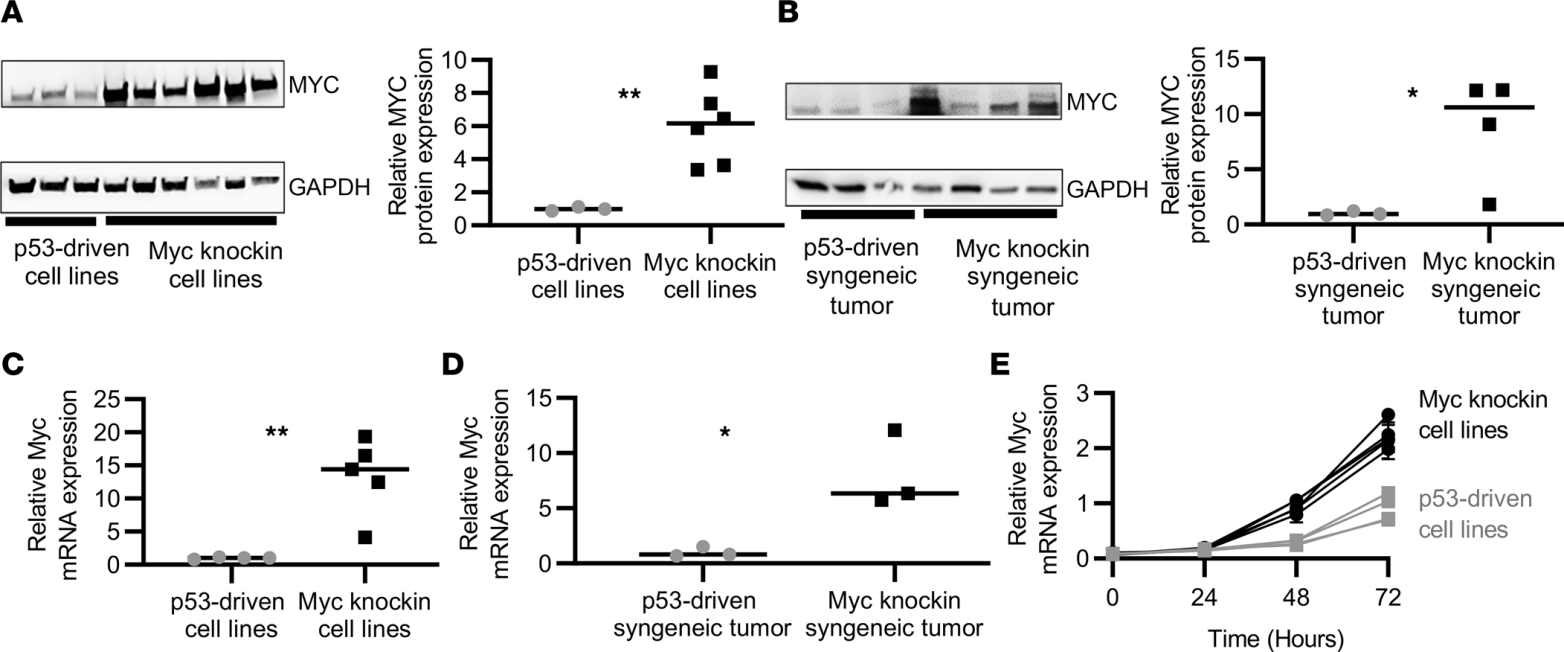
Proteotranscriptomic characterization of OS syngeneic mouse models and cell lines. (**A**) Western blot demonstrating increased MYC protein expression in *Myc*-knockin cell lines compared with p53-driven cell lines; quantified expression is shown in the right panel. (**B**) Western blot demonstrating increased MYC protein expression in *Myc*-knockin syngeneic mouse tumor tissue compared with p53-driven samples; quantified expression is shown in the right panel. (**C**) Quantitative PCR (qPCR) demonstrating increased *Myc* mRNA expression in *Myc*-knockin (*n* = 4) cell lines as compared with *Trp53^fl/+^* (*n* = 4) tumor cell lines. (**D**) qPCR demonstrating increased *Myc* mRNA expression in *Myc*-knockin syngeneic mouse (*n* = 4) compared with *Trp53^fl/+^* (*n* = 4) mouse. (**E**) *Myc*-knockin and p53-driven cell proliferation (the lighter line is for low-Myc cell lines and darker lines for high-Myc cell lines). (*P < 0.05,**P < 0.01.)

To develop resources for additional molecular and therapeutic studies, we generated and characterized GEMM-derived OS cell lines and syngeneic tumor models. Supplemental Figure 2A represents the schematic diagram of how the OS cell lines and the syngeneic mouse models were generated. Similar to the GEMM, the expression of MYC was significantly increased at the protein and mRNA levels in cell lines (*P* < 0.01 and *P* < 0.01) and syngeneic tumor models (*P* < 0.05 and *P* < 0.05) derived from the *Myc*-knockin as compared with the p53 tumors (Figure 2, A–D). The *Myc*-knockin cells were more proliferative than the p53-driven cells in vitro (Figure 2E). Further, we analyzed the MYC phosphorylation status at Ser62 position in *Myc*-knockin cell lines as it contributes to the stabilization of MYC protein. We observed that all *Myc*-knockin cell lines had phosphorylated Ser62 MYC (Supplemental Figure 2B). Other than Ser62 phosphorylation, threonine at amino acid position 58 also plays an important role in MYC protein stability as a phosphorylation site for subsequent ubiquitination recognition (23). This *Myc*-knockin model has the point mutation T58A; thus, it lacks any phosphorylation at that position, which has been previously demonstrated in another *Myc*-knockin model (24). We observed mice injected intra-tibially with *Myc*-knockin syngeneic cell lines rapidly developed tumors with a high incidence of metastatic disease like the spontaneous *Myc*-knockin GEMMs. Tumors were palpable 1–2 weeks after injection, and mice were sacrificed at roughly 3–4 weeks, with 60%–80% of syngeneic mice developing metastatic tumors primarily in the lung. In the case of p53-driven cell lines, those injected mice took approximately 2–3 weeks to develop a palpable tumor, and the time of sacrifice ranged from 6–12 weeks. We also noticed that the p53 cells injected in syngeneic mice were less metastatic in nature over the experimental time course, with only 10%–20% of the mice developing metastasis. These results demonstrate the aggressive nature of the *Myc*-knockin model and the utility of the syngeneic cell lines derived from the GEMM to recapitulate tumor development and progression, which can be used as valuable resources for downstream molecular and pharmacological studies.

### MYC suppresses immune cell infiltration to the TME in OS.

**Figure 3:**
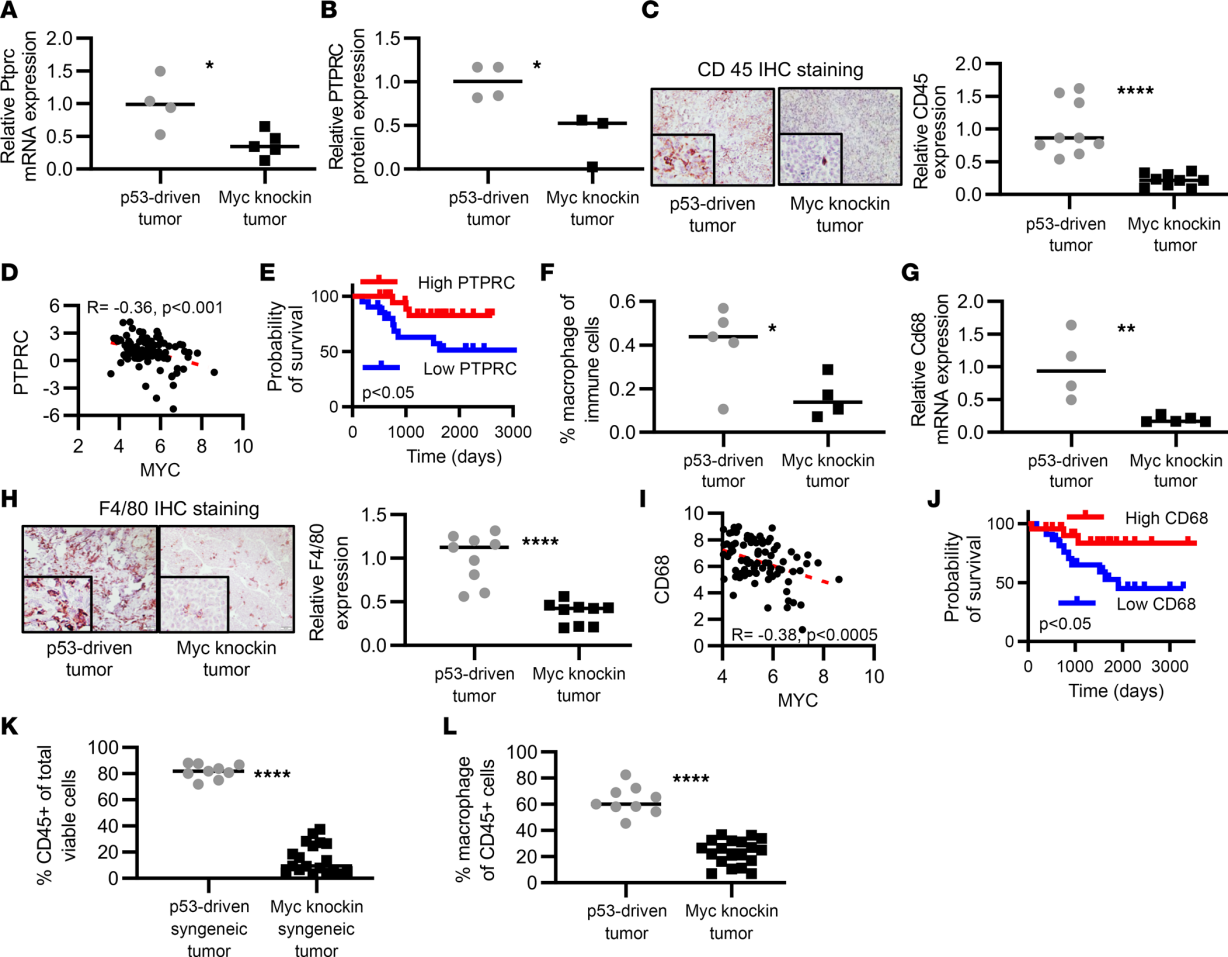
MYC suppresses immune cell infiltration into the OS TME. (**A**) Relative mRNA expression was analyzed by the RNA-Seq, demonstrating reduced *Ptprc* (*Cd45*) mRNA expression in *Myc*-knockin (*n* = 5) tumors compared with p53-driven (*n* = 4) tumor samples. (**B**) Protein expression analyzed by the total proteome analysis using mass spectroscopy demonstrating reduced PTPRC (CD45) protein in *Myc*-knockin tumors compared with p53- driven tumor sample. (**C**) IHC staining with CD45 in the paraffin-embedded tumor tissue samples showing lower expression in the *Myc*-knockin tumors (right panel) compared with p53-driven tumor (left panel) GEMMs. (**D**) Negative correlation between the *MYC* and *PTPRC* mRNA expression in human OS TARGET data set patients. (**E**) Kaplan-Meier curve of human OS TARGET data set for PTPRC expression with top quartile or bottom quartile samples. (**F**) Distribution of macrophage population in the tumor tissue sample of the *Myc*-knockin (*n* = 4) and p53-driven (*n* = 4) GEMMs analyzed by CIBERSORT. (**G**) Relative mRNA expression analyzed by RNA-Seq demonstrating reduced *Cd68* mRNA expression in *Myc*-knockin (*n* = 5) tumors compared with p53-driven (*n* = 4) tumor samples. (**H**) IHC staining with F4/80 (macrophage marker) in the paraffin-embedded GEMM tumor tissue samples showing lower expression in the *Myc*-knockin tumors (right panel) compared with the p53-driven tumor (left panel). IHC images were captured at 20× original magnification. Inset images are original magnification, 80×. (**I**) Negative correlation between the *MYC* and *CD68* mRNA expression in human OS TARGET data set patients. (**J**) Kaplan-Meier curve of human OS TARGET data set for *CD68* expression with top quartile or bottom quartile samples. (**K**) Relative immune cell populations (hematopoietic CD45^+^) in the syngeneic mouse tumor tissue samples analyzed by FACSymphony. (**L**) Relative macrophage populations (% of total CD45^+^ cells) in the syngeneic mouse tumor tissue samples analyzed by FACSymphony. *P < 0.05, **P < 0.01, ****P < 0.0001; log-rank (Mantel-Cox) test was performed for the Kaplan-Meier analyses.

GSEA of *Myc*-knockin and patient transcriptomic data identified that innate and adaptive antitumor immune response–related pathways were significantly downregulated in the high-MYC murine and human tumors as compared with low-MYC tumors (Supplemental Table 1). Specifically, we observed that the expression of *Ptprc* (CD45, a pan-hematopoietic cell marker) was significantly lower at the transcript (*P* < 0.05) and protein levels (*P* < 0.05) in the *Myc*-knockin tumors (Figure 3, A and B). IHC for CD45 on tumor tissue samples also showed a significant reduction in their expression in the *Myc*-knockin tumors as compared with the corresponding p53 tumors (Figure 3C).

To further validate our findings, we found a negative correlation between *MYC* and *PTPRC* mRNA expression in the human TARGET (*r* = –0.36, *P* < 0.001) and Kuijjer et al. (20) data sets (*r* = –0.25, *P* = 4.3 × 10^3^) (Figure 3D and Supplemental Figure 3A). In addition, high *PTPRC* mRNA expression was associated with a better prognosis compared with low *PTPRC* expression in the TARGET data set (*P* < 0.05) and trended toward significance in the Kuijjer data set (*P* = 0.059) (Figure 3E and Supplemental Figure 3B).

To further analyze the immune subpopulation, we performed a CIBERSORT analysis of our RNA-Seq data that gave us the fraction of 22 immunocyte types in OS tumor samples (Supplemental Figure 4). We observed a significant reduction in the macrophage population in the *Myc*-knockin tumor samples (*P* < 0.05) (Figure 3F). The expression of *Cd68* mRNA transcript, a macrophage marker, was also significantly decreased in the *Myc*-knockin GEMM tumor tissue samples as compared with the p53-driven tumors (*P* < 0.01) (Figure 3G). We validated this reduction of macrophage population in the TME of OS with IHC staining on paraffin-embedded GEMM tumor tissue samples using F4/80 antibody, which is a specific mouse macrophage marker (Figure 3H), which showed reduced macrophage population in the TME of *Myc*-knockin tumors. In addition, we observed that *Cd68* mRNA expression was negatively correlated with the *Myc* mRNA expression in the TARGET (*r* = –0.38, *P* < 0.0005) and Kuijjer data sets (*r* = –0.17, *P* = 0.05) (Figure 3I and Supplemental Figure 3C) and had prognostic significance, with higher *CD68* expression being associated with improved patient survival (Figure 3J and Supplemental Figure 3D). These findings not only further define a prominent role for macrophages in OS tumor biology but also validate our potentially novel murine *Myc*-knockin OS model for its ability to recapitulate human OS.

After identifying the direct association between elevated *Myc* expression and decreased immune infiltration, we applied FACS analysis on an orthotopic model using syngeneic murine high- and low-*MYC*-expressing cell lines to analyze the immune landscape of OS tumor (gating strategies shown in Supplemental Figure 5). The total CD45^+^ cells (hematopoietic cells) (*P* < 0.0001), as well as the macrophage population (*P* < 0.0001), were significantly lower in the *Myc*-knockin samples as compared with the p53-driven syngeneic tumor tissue samples (Figure 3, K and L). In summary, we identified that MYC regulates the OS TME by modulating immune cell populations, specifically macrophages. Additionally, macrophage-associated genes, *PTPRC* and *CD68*, are related to poor patient outcome.

### Myc hyperactivation downregulates the cytokine CSF1 in the TME of OS.

**Figure 4:**
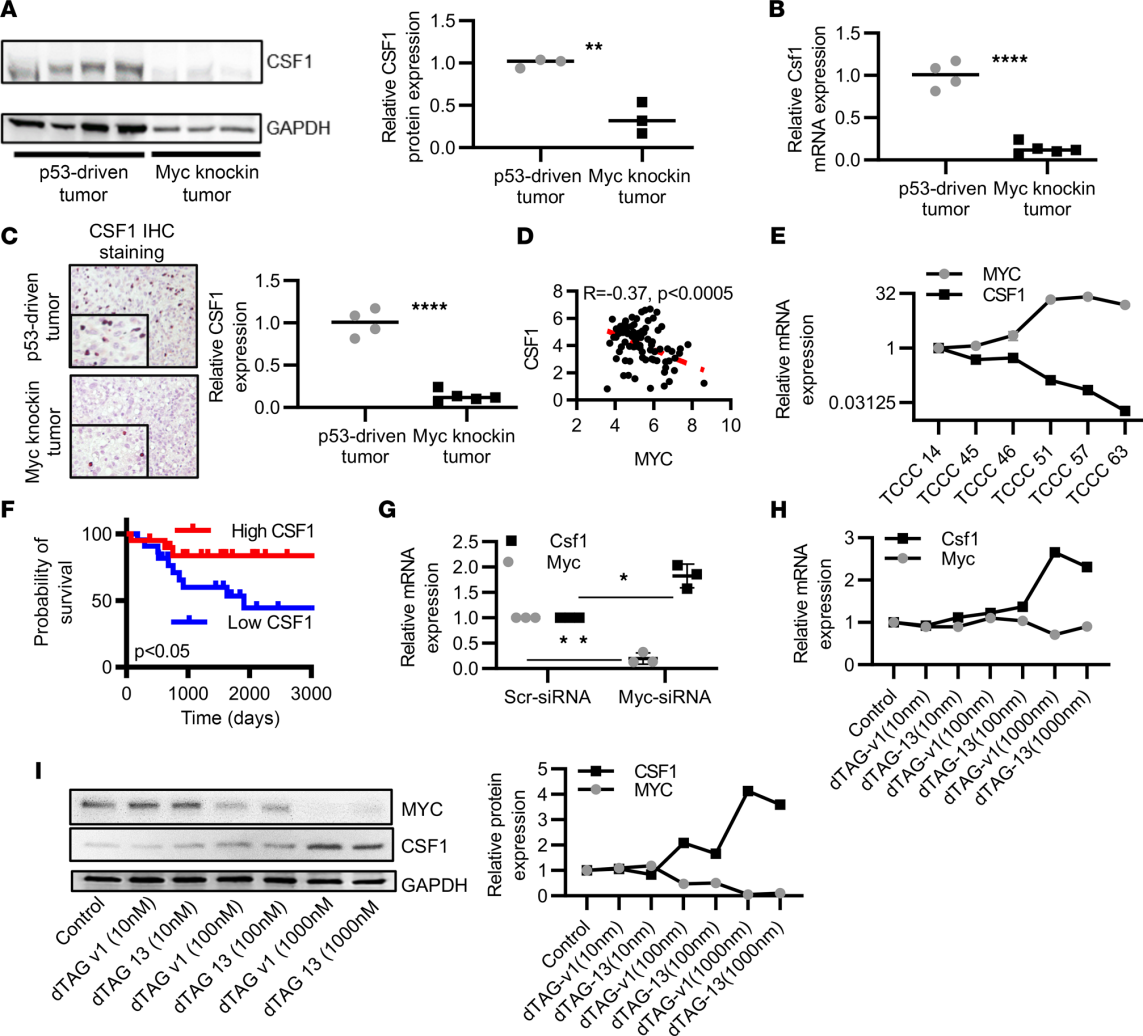
MYC association with CSF1 expression. (**A**) Western blot of CSF1 protein expression in *Myc*-knockin tumors compared with the p53-driven tumor. The quantified expression is shown in the right panel. (**B**) The RNA-Seq analyzed relative mRNA expression, demonstrating reduced *Csf1* mRNA expression in *Myc*-knockin (*n* = 5) tumors compared with the p53-driven (*n* = 4) tumor samples. (**C**) IHC staining for CSF1 in paraffin-embedded GEMM tumor tissue samples in the *Myc*-knockin tumors (lower panel) compared with p53-driven tumor (upper panel). The quantified expression is shown in the right panel. (**D**) *MYC* and *CSF1* mRNA expression in the PDX samples of the OS. (**E**) Negative correlation between the *MYC* and *CSF1* mRNA expression in human OS TARGET data set patients. (**F**) Kaplan-Meier curve of human OS TARGET data set for *CSF1* expression with top quartile (*n* = 22) or bottom quartile (*n* = 22) samples; log-rank (Mantel-Cox) test was performed for the Kaplan-Meier analyses. (**G**) *Csf1* expression upon transient knockdown of *Myc* in *Myc*-knockin murine OS cell lines. (**H**) *Csf1* mRNA expression after dTAG-13 and -v1 treatment. (**I**) Western blot of MYC and CSF1 protein expression after dTAG-13 and -v1 treatment; blot quantification is shown in the right panel. (*P < 0.05, **P < 0.01, ****P < 0.0001.)

To explore the mechanism underlying the alteration of the macrophage population in *Myc*-knockin OS TME, we investigated molecular alterations in macrophage maturation and recruitment. Our proteotranscriptomic analysis identified significant downregulation in the expression of *Csf1* in the *Myc*-knockin OS tumors as compared with the p53-driven tumor both at the protein (*P* < 0.01) and transcript levels (*P* < 0.0001) (Figure 4, A and B). Moreover, IHC staining for CSF1 validated lower protein expression in *Myc*-knockin GEMM tumors as compared with the p53-driven tumors (Figure 4C). To validate this finding in human samples, we investigated the correlation between *CSF1* and *MYC* mRNA expression in human OS tumors using the TARGET data set and available institutional patient-derived xenograft (PDX) samples. Interestingly, we observed a negative correlation between the *CSF1* and *MYC* expression for both the TARGET data set (*r* = –0.37, *P* < 0.0005) and the OS PDX samples (Figure 4, D and E). We also observed that a higher transcript expression level of *CSF1* was correlated with a good prognosis for patients with OS (Figure 4F). Besides *CSF1*, *IL-34* is an important ligand of CSF1R, so we further investigated the mRNA expression of *Il-34* in the *Myc*-knockin and p53-driven tumor tissue samples. We noticed there was no significant difference in the *Il-34* expression between the *Myc*-knockin and p53-driven tumor samples (Supplemental Figure 6), so we subsequently focused on the regulation and role of *Csf1* in hyperactivated MYC OS tumorigenesis.

To demonstrate that MYC modulates *CSF1* expression in OS, we used transient knockdown of the *Myc* transcript levels in *Myc*-knockin cell lines and the MYC–degradation tag (MYC-dTAG) protein degradation system using the murine F331 cell lines generated in our laboratory. The dTAG system allows real-time selective degradation of a target protein as a useful alternative to genetic methods for target validation. For the dTAG system, we stably expressed the FKBP12F36V-MYC^T58A^ construct in a low-*Myc*-expressing murine OS cell line (F331) described here as F331-dTAG-MYC cell line. Application of dTAG induced rapid, reversible, and selective degradation of FKBP12F36V-MYC^T58A^ fusion protein both in vitro and in vivo (Supplemental Figure 7).

After knockdown of the *Myc* transcript via Myc siRNA (si*Myc*), we observed a significant upregulation in the *Csf1* expression when compared with the corresponding scrambled control (*P* < 0.05) (Figure 4G). Furthermore, our complementary MYC-dTAG protein degradation model showed upregulation of *Csf1* upon exposure of the F331-dTAG-MYC cells to dTAG-v1 or dTAG-13 compounds. The *Csf1* expression level was substantially upregulated after the MYC degradation at the transcript and protein levels (Figure 4, H and I). Therefore, MYC negatively regulates the expression of CSF1. Subsequently, we were interested in investigating MYC-mediated mechanisms of CSF1 suppression. Additionally, to understand the role of MYC overexpression in OS regulating *Csf1r* expression in tumor-associated macrophages, we analyzed their expression at transcription and protein levels. CSF1R expression was significantly lower in *Myc*-knockin tumor compared with p53-driven tumor samples (Supplemental Figure 8, A and B). Subsequently, we performed coculture in vitro studies using RAW 264.7 cells, which are an established mouse macrophage cell line, and OS cells to assess the effect of MYC expression on expression of *Csf1r* in the population. We observed a significant reduction in the expression of *Csf1r* when RAW 264.7 cells were cultured with hyperactivated *Myc*-knockin cell lines compared with p53-driven cell lines (Supplemental Figure 8C).

### Myc represses Csf1 expression by empowering the miR-17/20a axis.

**Figure 5:**
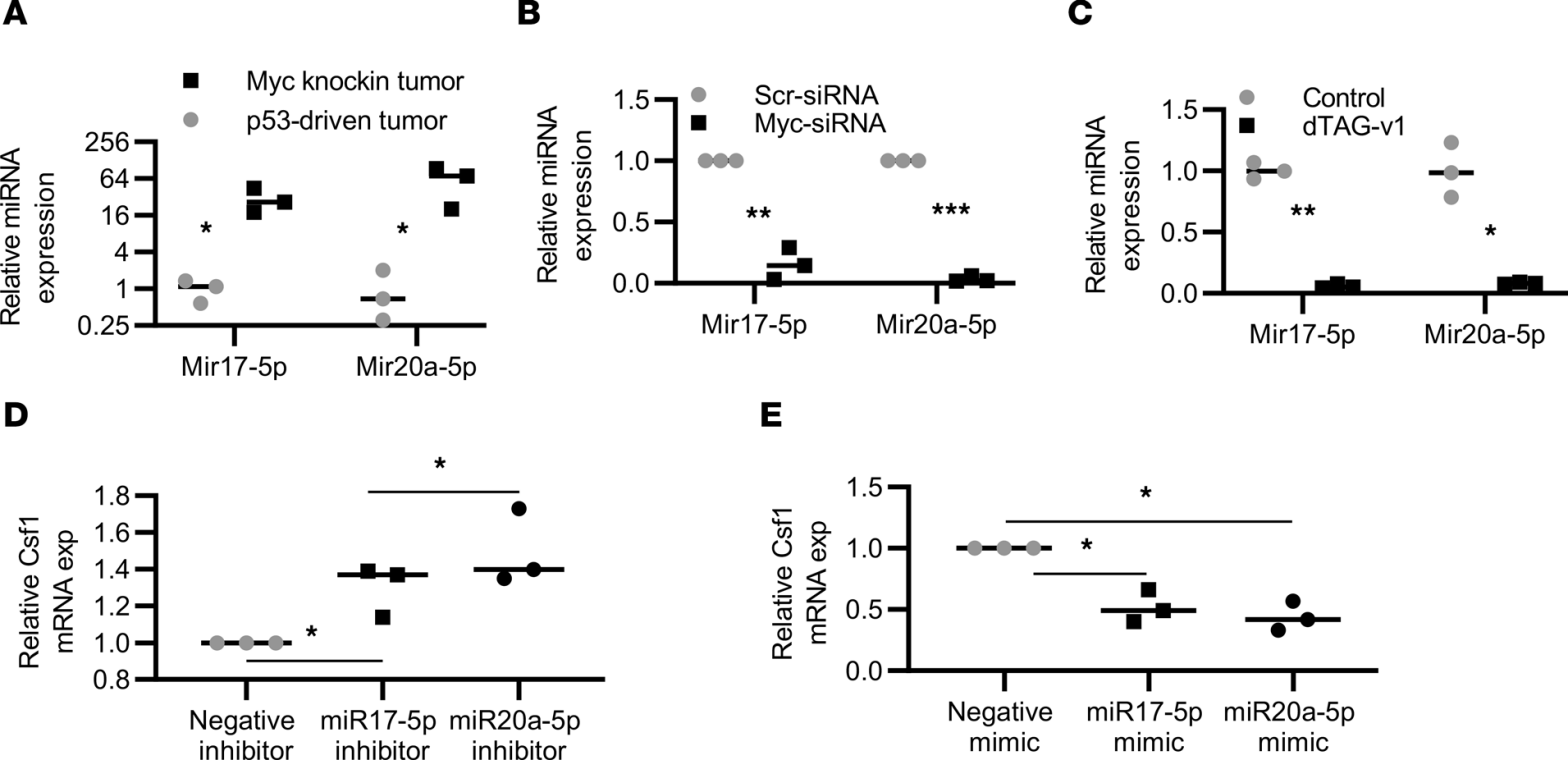
MYC represses *Csf1* expression through regulation of miR-17/20a. (**A**) qPCR for miR-17-5p and -20a-5p expression in *Myc*-knockin tumors and p53-driven GEMM tumor sample (*n* = 3). (**B**) qPCR for miR-17-5p and -20a-5p expression after *Myc* siRNA treatment compared with the Scr (siScr) control (*n* = 3). (**C**) miR-17-5p and -20a-5p expression after dTAG-v1 treatment (*n* = 3). (**D**) *Csf1* mRNA expression after miR17-5p and -20a inhibition treatment as compared with the negative inhibitor control (*n* = 3). (**E**) *Csf1* mRNA expression after miR17-5p and -20a mimic treatment compared with the negative mimic control (*n* = 3). (*P < 0.05, **P < 0.01, ***P < 0.001.)

To investigate the *Myc*-dependent mechanism of regulation of *Csf1* expression, we noted TargetScan predicted a binding region (1059–1065 nt) on *Csf1* mRNA for miR-17-92 family members (Supplemental Figure 9, A and B) (25), which are MYC-mediated miRs. MYC regulates the expression of several miRs, including the polycistronic miR-17-92 cluster, by binding to their promoter region in both humans and rodents (26–28). For a better understanding of miR-17-92 cluster activities, we focused on the function of miR-17 and miR-20a, 2 members of the miR-17-92 cluster. The expression of miR-17-5p and miR-20a-5p was significantly higher in the *Myc*-knockin compared with the p53-driven GEMM tumor tissue samples (*P* < 0.05) (Figure 5A). Further, we validated the MYC-dependent regulation of miR-17-5p/20a-5p expression using transient knockdown of MYC in mouse *Myc*-knockin cell lines and the MYC-dTAG protein degradation system using the F331-dTAG-MYC cells. The expression of miR-17-5p/miR-20a-5p after transient MYC knockdown and via the dTAG-MYC degradation resulted in the downregulation of miR-17-5p (*P* < 0.01 and *P* < 0.01) and miR-20a-5p (*P* < 0.001, 0.05) expression (Figure 5, B and C), thus validating a role for MYC in the regulation of miR-17-5p and miR-20a-5p expression in OS.

Further, to examine the role of miR-17-5p/20a-5p on *Csf1* regulation, we performed both gain- and loss-of-function studies using miR-17/20a inhibitors and mimics. *Myc*-knockin cell lines, which have significantly elevated levels of miR-17-5p/20a-5p expression, were treated with the inhibitors, whereas the p53-driven cell lines, in which miR-17-5p/20a-5p expression was lower, were used with mimic treatment. As shown in Figure 5D, after the treatment with miR-17-5p/20a-5p inhibitors, the expression of *Csf1* was significantly upregulated (*P* < 0.05), whereas miR-17/20a mimics reversed these effects and led to a downregulation of *Csf1* expression (*P* < 0.05) (Figure 5E). We established that miR-17-5p/20a-5p is responsible for at least part of the mechanism by which *MYC* regulates the *Csf1* expression in OS.

### Myc mitigates macrophage cell infiltration to the TME of OS and phenotypic function.

**Figure 6:**
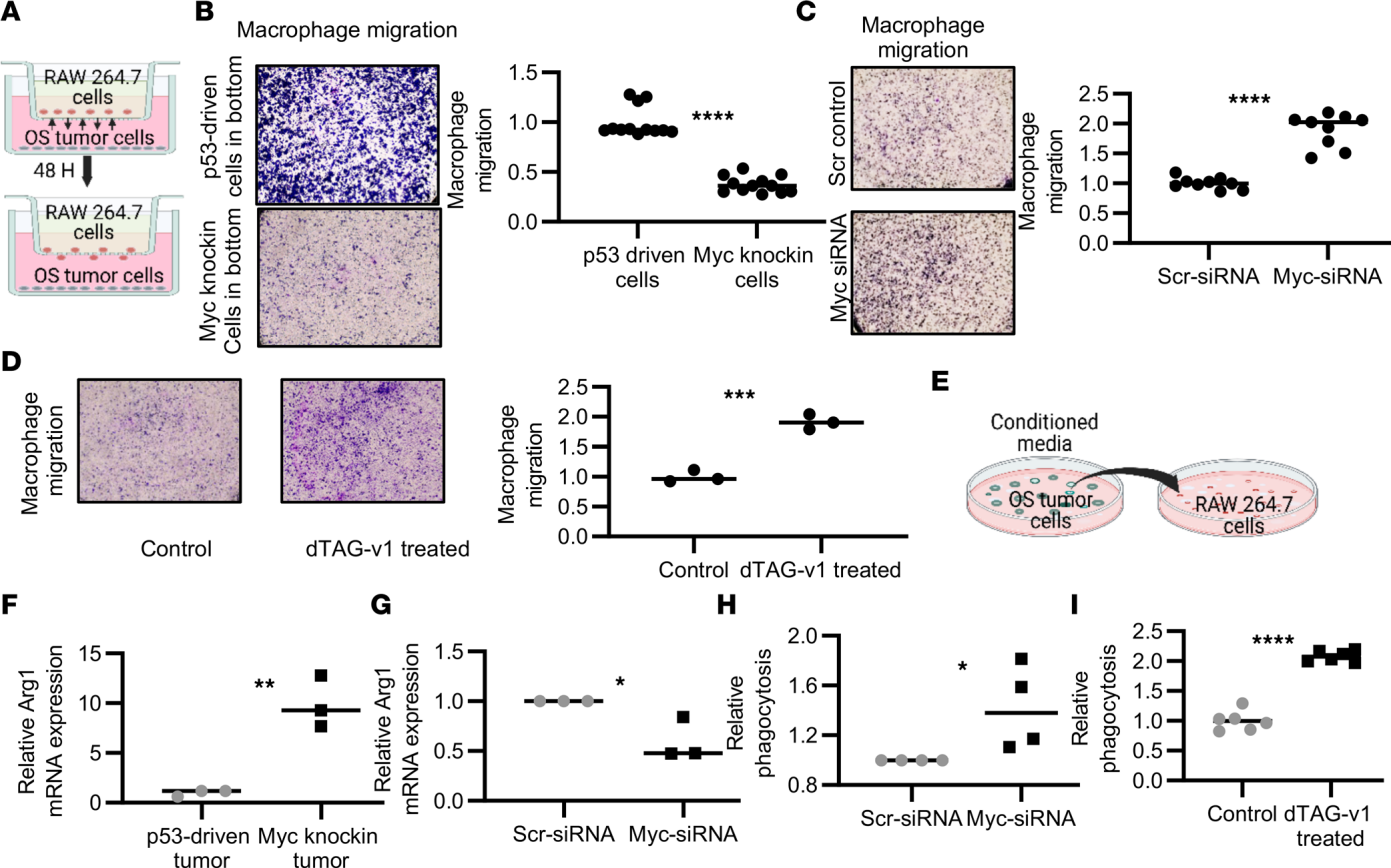
MYC mitigates intratumor macrophage cell infiltration and phenotypic function. (**A**) Schematic diagram showing the coculture setup. A total of 100,000 OS tumor cells were seeded on the bottom chamber, and 50,000 RAW 264.7 cells were seeded on the upper chamber and allowed to migrate for 48 hours. (**B**) Transwell migration of RAW 264.7 cells toward the *Myc*-knockin cell lines (upper panel) compared with the p53-driven cell line (lower panel); quantified RAW 264.7 cell migration is shown in the graph. (**C**) Migration of RAW 264.7 cells in the *Myc*-knockin cell line after si*Myc* (lower panel) as compared with the Scr control–treated (upper panel) OS cells; quantified RAW 264.7 cell migration is shown in the graph. (**D**) Migration of RAW 264.7 cells after dTAG-v1 treatment (right panel) compared with control (left panel) in the F331-dTAG-Myc cell line; quantified RAW 264.7 cell migration is shown in graph. (**E**) Schematic diagram showing the effect of cell culture supernatant on the RAW 264.7 cell polarization. (**F**) Expression of *Arg1* (M2 macrophage marker) in the RAW 264.7 cells cultured in the supernatant collected from the *Myc*-knockin cell lines (*n* = 3) as compared with p53-driven (*n* = 3) samples. (**G**) Expression of *Arg1* in RAW 264.7 cells cultured in the conditioned media after si*Myc* knockdown (*n* = 3). (**H** and **I**) Macrophage phagocytosis after si*Myc* and after MYC protein degradation (dTAG-v1). (*P < 0.05, **P < 0.01, ***P < 0.001, ****P < 0.0001.)

After identifying that the macrophage population was substantially diminished in the *Myc*-knockin OS tumors, we were interested in investigating the role of MYC in dictating this cellular microenvironmental feature. First, we examined the effects of elevated intrinsic MYC OS levels on the migration and proliferation of RAW 264.7 cells, using a Transwell coculture assay in an in vitro setup (Figure 6A). The OS cells, either *Myc* knockin or p53 driven, were cultured in the bottom chamber, and RAW 264.7 cells were seeded in the top chamber; the migratory potential of RAW 264.7 cells was monitored. In the wells with the *Myc*-knockin OS cells, we observed significantly lower amounts of macrophage migration compared with p53-driven OS cells (Figure 6B). To validate the role of MYC in this migration, we used 2 independent loss-of-function models, including si*Myc* and direct protein degradation via the dTAG system. A significant increase in the macrophage migration was observed after si*Myc* treatment compared with the scramble control in OS cells (Figure 6C). Migration was also increased after direct MYC protein degradation using the dTAG system (Figure 6D). These results demonstrate that MYC negatively regulates the macrophage cell infiltration to the TME of OS.

After validating the role of MYC in macrophage migration, we examined MYC’s involvement in dictating macrophage functions, particularly polarization and phagocytosis. To show the effect of MYC on the polarization of macrophage cells, we cultured RAW 264.7 cells in the conditioned media (CM) collected from the cell culture supernatants of the *Myc*-knockin and p53-driven cell lines (schematic diagram shown in Figure 6E). In a control experiment, RAW 264.7 cells were stimulated in vitro with cytokine pairs LPS and IFN-γ or IL-4 and IL-13 to transform them into the M1- and M2-like macrophage subpopulations, respectively. The morphology of the transformed cells was analyzed, and the M1- and M2-related genes (*Cd86* and arginase 1 [*Arg1*], respectively) were quantified at the transcriptional level (Supplemental Figure 10, A–C). We observed an upregulation in the expression of *Arg1* in the RAW 264.7 cells cultured in the *Myc*-knockin cell line CM compared with the p53-driven cell lines (Figure 6F). Moreover, we observed similar gene expression changes when we treated the RAW 264.7 cells with IL-4 and IL-13 cytokines (Supplemental Figure 10B). *Cd86* expression was not significantly different when it was compared between the experimental groups (Supplemental Figure 10D). To verify the MYC-dependent macrophage transformation, we cultured RAW 264.7 cells in the CM collected from the si*Myc*-knockdown OS cell culture supernatant and compared gene expression with that of the siScr control cell culture. A significant reduction in the *Arg1* expression (Figure 6G) and enhancement in the *Cd86* were observed in the RAW 264.7 cells cultured in the MYC-knockdown OS supernatant culture (Supplemental Figure 10E).

Last, we analyzed if enhanced MYC expression in OS affects the phagocytic nature of macrophage cells. To establish the role of *Myc* in macrophage phagocytosis, we used transient gene and protein knockdown via si*Myc* and the dTAG protein degradation system. RAW 264.7 cells were cultured in the CM collected from *Myc*-knockin cell lines treated with si*Myc* or siScr control. RAW 264.7 cells were cultured in the CM collected from either dTAG-v1– or DMSO-treated F331-dTAG-MYC cells. A significant enhancement in the phagocytosis was observed for the RAW 264.7 cells cultured in the CM from si*Myc*-treated (*P* < 0.05) and dTAG-v1–treated (*P* < 0.0001) cells compared with the corresponding controls (Figure 6, H and I).

In addition, we determined if MYC-regulated CSF1 modulates macrophage functions including their migration, phagocytosis, and proliferation in the OS TME. To examine the role of MYC-mediated CSF1 in macrophage cell migration, we used a Transwell coculture model (Figure 6A). As shown in Supplemental Figure 11A, after transient knockdown of *Csf1* expression in OS cells, we observed a significant reduction in the RAW 264.7 cell migration toward the OS cells compared with the scramble control. Our p53-derived cell lines were used for the *Csf1*-knockdown experiment as their expression was relatively higher. After validating the role of CSF1 in the RAW 264.7 cell migration, we performed a rescue experiment using the F331-dTAG-MYC cell line to determine the role of MYC-dependent CSF1 in macrophage cell migration. Specifically, we used the dTAG-v1 to degrade the MYC protein followed by transient knockdown of the *Csf1* gene to examine their role in macrophage cell migration. Supplemental Figure 11B shows after dTAG-v1 treatment, migration of macrophages increased and later decreased followed by the *Csf1* knockdown. We also observed that macrophage proliferation was significantly enhanced in the presence of CSF1 (Supplemental Figure 11C). We conclude that MYC is sufficient to regulate the CSF1 expression in the OS tumor, which orchestrates the migration of macrophages in the TME of OS.

In summary, we demonstrated that intratumor MYC dictates environmental macrophage cell migration and functions, particularly polarization and phagocytic properties, in the TME of OS.

### Selective in vivo pharmacological degradation of Myc improves immune cell infiltration to the TME of OS.

**Figure 7:**
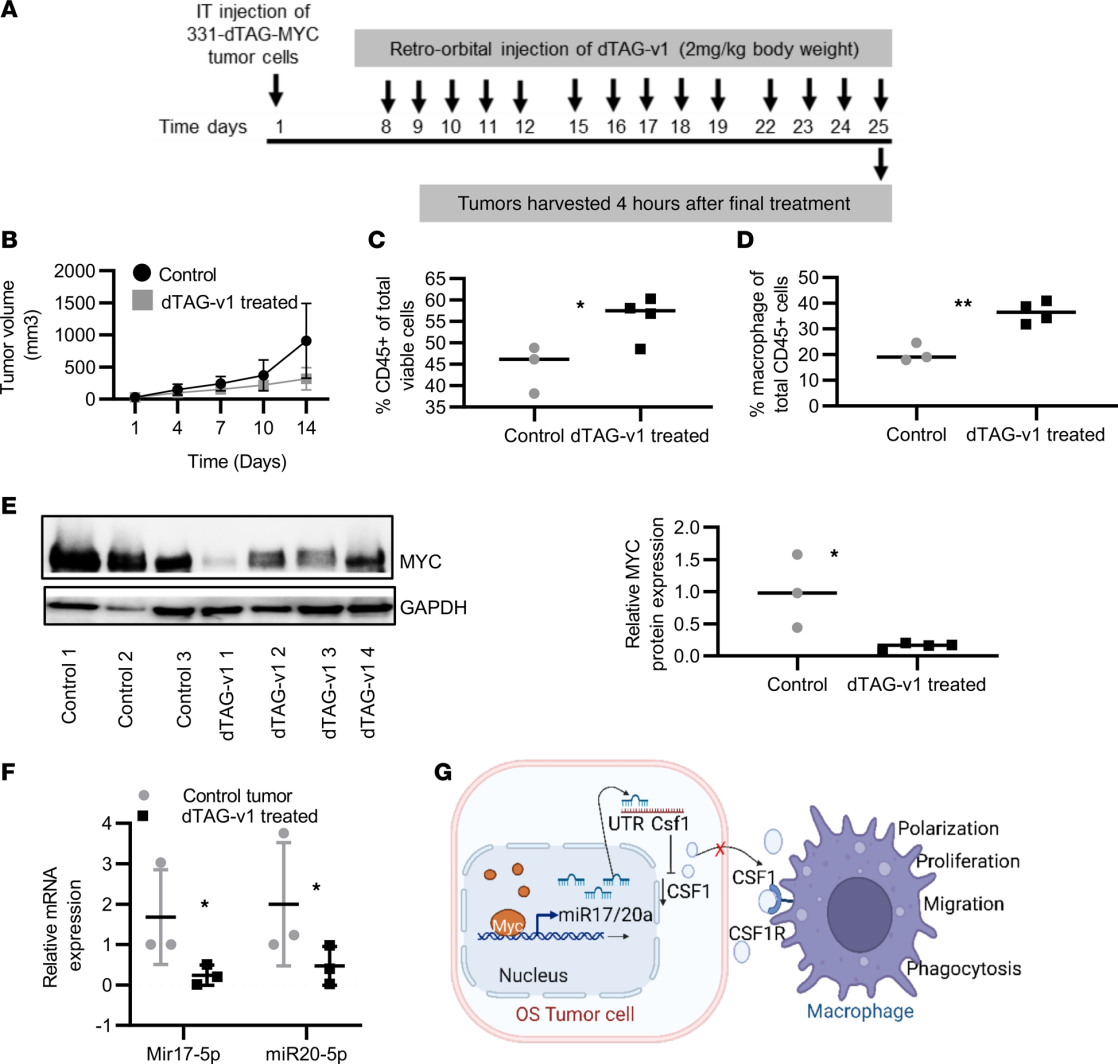
Selective in vivo pharmacological degradation of MYC protein levels and its effects on the OS immune landscape. (**A**) Schematic diagram showing the tumor cell injection and dTAG-v1 drug treatment. (**B**) Tumor volume after 2 weeks of control (*n* = 3) and dTAG-v1 treatment (*n* = 4). (**C**) Analysis of intratumor FACS analysis for CD45 population after control (*n* = 3) and dTAG-v1 treatment (*n* = 4). (**D**) Macrophage population analysis after 2 weeks of dTAG-v1 treatment. (**E**) Western blot analysis for MYC expression after 2 weeks of the dTAG-v1 treatment. Blot quantification data are shown in right panel. (**F**) Expression of miR-17/20a after control and dTAG-v1 treatment (*n* = 3/cohort). (**G**) Schematic diagram showing the MYC-dependent regulation in the TME of OS. (*P < 0.05, **P < 0.01.)

To validate the MYC-dependent regulation of the immune infiltration and macrophage functions, we used the dTAG protein degradation approach in vivo. F331-dTAG-MYC cells were injected intra-tibially into C57BL/6 mice, and upon detection of palpable tumor, mice were randomized to receive treatment either with dTAG-v1 or with vehicle control for 2 weeks intravenously via retro-orbital injection. Figure 7A shows the schematic diagram for the dTAG-v1 treatment. Tumor volume was significantly reduced after 2 weeks of dTAG-v1 treatment when compared with the placebo control group (Figure 7B). As shown in Figure 7C, after 2 weeks of treatment, overall CD45^+^ cell populations were significantly enhanced in the dTAG-v1–treated group as compared with the placebo control. The macrophage population was also significantly enhanced after the treatment compared with the placebo control group (Figure 7D). Effective MYC protein degradation was noticed after dTAG-v1 treatment (Figure 7E). The analysis of miR-17/20a transcript expression showed a significant reduction after the dTAG-v1 treatment (Figure 7F). Together, these data support the involvement of MYC in the immune-suppressive TME in OS by regulating the macrophage population (Figure 7G).

## Discussion

Despite surgical advancements and multidrug systemic chemotherapy, the overall survival for patients with OS has had minimal improvements over the last 3 decades (29). The rarity of this disease, the tumor cell heterogeneity, and the lack of targetable robust oncogenic mutations make it very challenging to study and cure a significant number of patients with OS. While there is a lack of identifiable targetable mutations, OS has recurrent chromosomal copy number alterations encompassing gains and losses of key oncogenes or tumor suppressor genes. Amplification of c-MYC is seen in a significant portion of OS tumors and conveys an overall poor prognosis. We have established and characterized a potentially novel conditional osteoblast-specific *Myc*-knockin GEMM to understand the pathophysiology of OS tumors and the role of MYC in the TME modulation. Moreover, we demonstrated the critical role of MYC in regulating macrophages in the TME and its impact on the generation of more aggressive tumors.

MYC oncogene is considered a master regulator of many processes, including cell cycle entry, ribosome biogenesis, and metabolism, and its expression is dysregulated in more than half of human cancers (10, 30). We previously generated a conditional osteoblast-specific OS GEMM by altering the *Trp53* status (21, 31). However, the *Trp53*^fl***/***+^ *Myc*-knockin GEMM we have generated and comprehensively characterized has high *Myc* expression at the mRNA and protein levels with accelerated osteosarcomagenesis and metastatic potential compared with the previously reported *Trp53^fl/+^* OS GEMM model (21). We subsequently generated murine OS cell lines and applied them toward orthotopic syngeneic tumor models for additional molecular and therapeutic studies. Our potentially novel *Myc*-knockin OS GEMM closely resembles the high-*MYC*-expressing human OS subtype and has significant homology to genomic and biological phenotypes seen in these tumors. Through the integration of innovative murine models of OS and bioinformatics analysis of human OS data sets, we have identified a likely novel immune-regulatory function of MYC in OS tumor biology.

We established that hyperactivation of MYC suppresses immune cell infiltration, including macrophages in the OS TME. Recent studies elucidate the role of MYC in TME modulation as well as in the host immune response in multiple tumor types (14, 32, 33). The macrophage has been reported to be the most abundant immune cell infiltrate to the TME of solid tumors, including OS (34), and is involved in regulating other immune cell functions and matrix remodeling that leads to tumor-suppressing or -promoting microenvironments (35, 36). Recently, Lee et al. reported similar findings in the triple-negative breast cancer tumor, where they found elevated *MYC* expression was associated with lower overall immune cell infiltration, including the macrophages in the mouse models and patient data (37). MYC has been found to be associated with immune-suppressive TME in lung and pancreatic cancer models (32).

The role of a macrophage in tumor progression for OS remains to be fully elucidated, in part due to the contrasting roles they play depending on their polarization. The excessive macrophage infiltration in the TME and its association with the patient’s clinical outcome depend on the cancer diagnosis. The abundance of macrophages in the TME of colorectal and gastric cancer is associated with a good prognosis whereas macrophage abundance in breast, head and neck, glioma, melanoma, and prostate cancers confers the worst prognosis (38–40). Macrophages are highly plastic cells and can polarize into different subpopulations, such as M1- or M2-like macrophages, depending on the microenvironmental signals in the TME. On the one hand, M2-like macrophages promote the immune-suppressive TME by recruiting regulatory T cells, inhibiting the T cell function by controlling the expression of programmed cell death ligand 1 and cytokines IL-10 and TGF-β (41, 42), and inhibiting macrophage phagocytosis by upregulating the expression of sirtuin (43). On the other hand, M1-like macrophages take part in the adaptive anticancer immune response by enhancing antigen presentation, activating adaptive immunity, and enhancing phagocytosis (35). We noted an inverse correlation between the hyperactivation of *Myc* and the abundance of macrophage populations in the TME of OS of our murine model. The human TARGET and R2 data sets also show a negative enrichment of the macrophage population in the abundance of MYC expression. Additionally, the higher macrophage population was associated with the OS patient’s good prognosis. Similar to our finding, the abundance of macrophages was associated with reduced metastasis and improved survival in high-grade OS (44). However, it should be noted that the overall macrophage population and patient outcome depend on the tumor type. The levels of *CD68*, a macrophage marker, correlated with an adverse prognosis in glioblastoma, kidney renal clear cell carcinoma, hepatocellular carcinoma, lung squamous cell carcinoma, and thyroid carcinoma and a favorable prognosis in colorectal cancer, OS, and kidney chromophobe (44–49). We think the heterogeneity of phenotypes is behind the inconsistency of these functions in various cancer types.

As CSF1/CSF1R has been identified as the principal pathway that controls macrophage survival and differentiation from progenitor or circulating monocytes to macrophages (50, 51), we examined the role of CSF1 in MYC-associated macrophage regulation in the TME of OS and identified a potentially novel MYC*/*miR-17-92/CSF1 axis that directly contributes to this alteration in the TME. MYC regulates the miR-17/20a expression by directly binding to their UTR (28, 52). The miR-17-92 family is overexpressed in various human cancers, including lung, breast, colon, B cell lymphoma, gastric, and retinoblastoma, where they regulate several genes important for cell cycle progression and metastasis (53–56).

Furthermore, we demonstrated MYC-dependent macrophage polarization, phagocytosis, and migration to the TME of OS. Currently, 2 macrophage-centered approaches are in clinical trials. One includes eliminating tumor-associated macrophages and the other repolarizing tumor-promoting macrophages into pro-inflammatory M1-like macrophages. The blocking of the CSF1/CSF1R axis by targeting CSF1 or CSF1R is in clinical trials to eliminate the tumor-promoting macrophage population (57). But the results are contradictory; in a recent breast cancer clinical trial, the use of neutralizing anti-CSF1R and anti-CSF1 antibodies, along with the small-molecule inhibitors of CSF1R, showed an enhancement in the metastasis without altering primary tumor growth (58). Another study observed a strong correlation between the clinical chemotherapy response and a higher expression of CD4/CD68/CSF1R gene signatures in OS (59). These results indicate that the role of the CSF1/CSF1R system is far more complex than it seems and requires further investigation as a therapeutic target.

CSF1 regulates macrophage proliferation, function, and infiltration to the TME. The association of the macrophage population abundance with the disease prognosis dramatically depends on the tumor subtypes, so targeting the CSF1/CSF1R pathway may vary greatly depending on the tumor subtypes. So, before targeting the macrophage or CSF1/CSF1R pathway, one must consider the tumor subtype and its genetic drivers. We noticed in the case of *Myc*-knockin OS that CSF1 expression was low and had a more aggressive and metastatic tumor. So, targeting CSF1 might not be a rational approach in this subpopulation of patients. The other approach, which might be clinically beneficial for patients with OS and for effective therapy development, is the use of drugs that can polarize the M2-like macrophage population to M1 macrophages. Based on the available preclinical and clinical data, for example, mifamurtide showed some benefits in OS treatment and has approval from the European Medicines Agency, but further investigations are required to define its role in the treatment of patients with OS (60). Other potential molecules that could be used to transform the M2-like macrophage phenotype to M1-like phenotypes include linear 3-O-methylated galactan, CD40 agonist, zoledronic acid, statins, trabectedin, and TLR ligands (e.g., imiquimod and CpG) (61–64). But all these approaches require additional investigation before being used in the clinical setup.

We and others think that innate immune cells, especially macrophages, play an essential role in inhibiting the initiation and development of cancer (35), and reorienting and polarizing tumor-associated macrophages toward the M1-like macrophage is the holy grail of macrophage-mediated cancer therapy (65, 66). Our preclinical mouse model will help in screening the small molecules including the immune modulators, macrophage polarizers, and a combination of drugs to target this aggressive tumor subtype and provide more insight into the OS tumor biology. Future studies also include further dissecting the role of macrophages in MYC-driven OS by evaluating the cell-cell communication between the macrophage and OS cells as well as modulation of the macrophage population. In addition, additional mechanistic studies will assess the effects of stably altering miR-17/92 expression in OS cells to determine the in vivo effects of these miRs. Presently, systemic administration of anti-miR therapy is very exploratory, and results can often be difficult to interpret or use to make definitive conclusions.

Overall, our study is the first to our knowledge to successfully demonstrate a MYC-dependent regulation of the OS TME. We have identified a likely novel molecular mechanism in which MYC hyperactivation leads to the downregulation of *Csf1* through increased miR-17-92 expression, resulting in diminished macrophage presence in the TME of OS. Thus, perturbations of direct MYC activity, or downstream effectors, such as miR-17/92 family members, can have potent effects on enhancing the tumor immune microenvironment and therapeutic benefit for this high-risk group of patients with OS.

Our studies using a c-*Myc*–knockin OS GEMM and cell line models identified that hyperactivation of c-MYC is sufficient to inhibit the CSF1 expression associated with the macrophage proliferation and localization to the TME of OS. We established the involvement of MYC-dependent miR-17/20a in the regulation of *Csf1* resulted in those phenotypes. Thus, we identified a potentially novel molecular mechanism through which MYC regulates the CSF1 expression. This leads to alterations in the macrophage population and an immunosuppressive TME.

## Methods

### Generation of Myc-knockin GEMM.

*Myc*-knockin GEMM was generated by crossing Col2.3-Cre *Trp53^fl/+^*and Lox-Stop-Lox-*Myc^T58A^* mice, which were developed and obtained from the Sears lab (Oregon Health & Science University) (24). The Col2.3-Cre *Trp53^fl/+^* mouse model was generated by crossing mice expressing Cre recombinase under the transcriptional regulation of the osteoblast-specific promoter Col2.3 with *Trp53*-floxed mice and represented as p53-driven mice (21). Genotyping primer details are given in Supplemental Table 2A. The IACUC approved the experimental protocol (AN-5225). All experiments were performed per relevant guidelines and regulations.

### Generation of an OS syngeneic mouse model and cell lines.

Primary murine tumor OS cell lines were generated by dissociating the GEMM OS tumor using Miltenyi Biotec Tumor Dissociation Kit (catalog 130-096-730). OS cell lines were cultured in DMEM supplemented with 10% FBS and 1% pen/strep and maintained at 37°C with 5% CO_2_. All cell lines were routinely checked for Mycoplasma contamination. Alkaline phosphatase staining (NBT/BCIP, catalog no-11697471001, Roche) was performed according to the manufacturer’s instruction to confirm the OS cell type. A syngeneic mouse model was generated by injecting 1 × 10^6^ OS tumor cells intra-tibially into C57BL/6 mice (The Jackson Laboratory).

### Cell proliferation assays.

Growth assays were performed by plating 1,000 cells per well in a 96-well dish. Cell growth was assessed daily by the addition of the Cell Counting Kit-8 (CCK-8) reagent, according to the manufacturer’s instructions (CCK-8 assay kit; Dojindo Laboratories). Each cell line was plated in triplicate, and the value presented represents the average of the samples.

### Transfection of siRNA in OS cell lines.

In vitro transfections were performed in 6-well plates (2 × 10^5^ cells) for OS cells derived from the *Myc*-knockin and p53-driven GEMMs, using Lipofectamine RNAiMAX (Invitrogen) as recommended by the manufacturer. For the transfection experiments, cells were plated 24 hours before the experiment. For each candidate gene, 2 predesigned gene-specific siRNAs (MilliporeSigma) were tested in parallel with scrambled control (MilliporeSigma) as well as a blank with the transfection agent only. The most effective siRNAs for *Myc* and *Csf1* were selected (catalog SASI_Mm01_00157478 and SASI_Mm02_00308073, MilliporeSigma) for further experiments. Cells for mRNA evaluation were harvested 48 hours posttreatment, while those for protein evaluation were harvested at 72 hours.

### Quantitative real-time PCR.

Total mRNA and miR were extracted by using the RNeasy Mini Kit (QIAGEN) and miRNeasy Mini Kit (QIAGEN), respectively, as recommended by the manufacturer. In brief, after removing the medium, cells were washed twice with cold PBS and lysed in RLT lysis buffer (QIAGEN) for 5 minutes at room temperature. For tumor tissue samples, around 30 mg of tumor sample was homogenized in either triazole for miR isolation or RLT lysis buffer for total RNA isolation using the tissue homogenizer. Lysates were stored at –20°C before the RNA isolation. RNAs were quantified using Thermo Fisher Scientific NanoDrop 2000. We used 1 μg RNA for cDNA synthesis using qScript cDNA SuperMix (Quantabio). Real-time PCR with iQ SYBR Green SuperMix (Bio-Rad) was performed using gene-specific primer pairs (QIAGEN/MilliporeSigma) utilizing the StepOnePlus real-time PCR machine (Applied Biosystems). The hsa-miR-17-5p and 20a-5p miRCURY LNA miRNA PCR Assay Kits were used to quantify the miR expression. The relative mRNA expression was calculated with the ΔΔCT method. A list of primers is given in Supplemental Table 2B.

### Western blot analysis.

After removing the medium, cells were washed twice with cold PBS and lysed in ice-cold RIPA lysis buffer (50 mM Tris-Cl, pH 7.4; 150 mM NaCl; 1% NP-40; 0.25% Na-deoxycholate) supplemented with protease inhibitors and phosphatase inhibitors (Roche) for 15 minutes at 4°C. Lysates were harvested and centrifuged at 13,000*g* for 10 minutes to remove the cell debris, the supernatant was collected, and the protein concentration was determined using the bicinchoninic acid assay (Pierce) and stored at –20°C before the analysis. For tumor tissue samples, around 50 mg of tumor sample was homogenized in the RIPA lysis buffer using the tissue homogenizer, and the supernatant was collected similarly as described above for the cell lines. A total of 30 μg of protein was electrophoresed in a 4%–15% precast gel (Mini-PROTEAN TGX) SDS-PAGE under reducing conditions. iBlot 2 dry blotting system was used for transferring the protein to the PVDF membrane. After blocking the blot with 5% BSA in PBS, the blots were probed overnight with antibodies (c-MYC 1:1,000, ab32072, Abcam; CSF1 1:1,000, ab233387, Abcam; phospho-cMYC Ser62 1:1,000, 13748, Cell Signaling Technology; GAPDH 1:1,000, AB2302, MilliporeSigma). Blots were incubated with the appropriate secondary HRP-conjugated antibodies for 1 hour, and the signal was detected utilizing MilliporeSigma Immobilon Western chemiluminescent HRP substrate. Quantification and statistics Western blots were visualized and quantified using ImageJ software version 1.53e (NIH). Statistical significance was determined by Student’s 2-tailed *t* test.

### IHC.

Primary as well as metastatic tumor tissue samples from the *Myc*-knockin GEMM were fixed in 10% formalin at the time of tumor harvest, paraffin-processed, and sectioned. For IHC analysis, sections were deparaffinized, rehydrated, and boiled in a microwave for 20 minutes in 10 mM citrate buffer (pH 6.0) for antigen retrieval. VECTASTAIN Elite ABC kit (Vector Laboratories) following the manufacturer’s instructions was used for visualization. For control, IgG isotype (Thermo Fisher Scientific catalog 02-6102) was used instead of primary antibody, wherever indicated. Tumor sections were stained with hematoxylin and eosin and analyzed with the help of Baylor core pathologists for OS tumor characterization. IHC slides were visualized using the phase-contrast microscope (BX53 Biological Microscope, Olympus).

### SymphonyFACS analysis.

Tumors were excised from the syngeneic mice and dissociated using the gentleMACS Dissociator (Miltenyi Biotec) according to the manufacturer’s protocol. Cells were passed through the 70 μm cell strainer to remove cell clumps. RBCs were lysed using 1× RBC lysis buffer (eBioscience, Thermo Fisher Scientific, catalog 00-4300-54). After centrifugation at 200*g* for 5 minutes, the supernatant was discarded, and cells were resuspended into 1 mL of FACS buffer (PBS with 5% FBS) followed by the staining with 0.1% fixable viability stain (BD Biosciences, catalog 565388) along with 10 μL of brilliant stain buffer plus (catalog 566385) for 10 minutes at 4°C. Cells were surface-stained with a premixed fluorescence conjugated mAb cocktail for 30 minutes at 4°C in the dark. The cocktails were prepared with CD45-BUV805 (catalog 748370), F4/80-BV 421 (catalog 565411), CD11B PE-CF594 (catalog 562287), CD3 BV711 (catalog 740665), CD19 BUV 395 (catalog 565965), and Siglec-F PE (catalog 552126). After washing, cells were fixed in 2% PFA for 30 minutes at 4°C and finally resuspended in 0.5 mL of FACS buffer and stored at 4°C before analysis. Data were acquired on 5-laser FACSymphony (BD FACSymphony A5 Cell Analyzer). Analysis was performed using BD FACSDiva software v. 6.0 and FlowJo 10.8.0 (Tree Star, Inc). Gating strategies for immune cells were used as follows: total leukocytes CD45^+^, macrophages CD45^+^CD11B^+^F4/80^+^.

### dTAG protein degradation system.

A Nobel degradation tag (dTAG) system was used to selectively target the MYC protein degradation. The dTAG system provides a linker that links FKBP12-F36V fused with MYC protein to ubiquitin ligase and then degrades it through the ubiquitin-proteasome system as described previously by Nabet and Roberts (67). We have generated a syngeneic murine OS cell line (F331-dTAG-MYC), which was originally derived from a Col2.3-Cre/*Trp53^fl/+^* GEMM tumor, with a low level of endogenous c-MYC, but stable overexpression of the FKBP12F36V-MYC^T58A^ construct (stable cell line production courtesy of Tong Liang and Charles Lin, Baylor College of Medicine). Commercially available dTAG-v1 (Tocris, Bio-Techne Corporation, catalog 6914) and dTAG-13 (Tocris, Bio-Techne Corporation, catalog 6605) at a concentration range from 10 nM to 1 μM were used to selectively degrade MYC protein.

### In vivo MYC protein degradation.

We injected 1 × 10^6^ viable F331-dTAG-MYC cells expressing FKBP12F36V-MYC^T58A^ into 4-week-old C57BL/6 mice through intra-tibial injection. Before injection, cells were tested for Mycoplasma contamination. Mice were routinely monitored for the tumor and after the confirmation treated for 2 weeks either with the vehicle control (*n* = 3 biologically independent mice) or with dTAG-v1 (2 mg/kg, *n* = 4 biologically independent mice) through the retro-orbital injection. The dTAG-v1 was formulated in 20% solutol (MilliporeSigma), 5% DMSO, in 0.9% sterile saline. After 2 weeks of treatment, tumors were harvested and dissociated for the FACSymphony analysis using the gentleMACS dissociator (Miltenyi Biotec). Protein and RNA samples were prepared from the tumor samples and stored at –80°C before analysis. All experiments were adherent to institutional standards.

### Tumor-conditioned media collection.

Both *Myc*-knockin and p53-driven OS cell lines were cultured separately in DMEM supplemented with 10% FBS and 1% pen/strep. Once grown to 90% confluence, media were discarded and rinsed with PBS. Cells were then incubated with fresh DMEM without any supplement for 24 hours; the CM were collected and centrifuged at 200*g* for 5 minutes to remove cell debris. The supernatant was filtered with a 0.20 μm syringe filter and stored at −20°C before use. We also collected CM from the *Myc* and *Csf1* siRNA–knockdown cell lines as well as the corresponding Scr control cells.

### Coculture experiment.

The GFP-labeled murine macrophage cells, RAW 264.7 (gifted by Ananth V. Annapragada, Texas Children’s Hospital, Baylor College of Medicine), were cocultured with the murine OS cells in DMEM supplemented with 10% FBS and 1% of pen/strep and maintained in a humidified atmosphere with 5% CO_2_ at 37°C. Before coculture, RAW 264.7 cells were transiently transfected with GFP plasmid (Addgene plasmid 176015), and GFP-positive cells were FACS-sorted using the BD FACSAria II Cell Sorter. *Myc*-knockin and p53-driven OS cells were cocultured separately with GFP-labeled RAW 264.7 cells at a ratio of 1:4 (RAW 264.7/tumor cells) for 72 hours. After coculture, GFP-labeled-RAW 264.7 cells were FACS-sorted by BD FACSAria II Cell Sorter, and *Csf1R* mRNA expression was analyzed in the RAW 264.7 cells using semiquantitative PCR.

### Monocyte differentiation to M1/M2-like macrophages.

An established mouse macrophage cell line, RAW 264.7, was used for the polarization/differentiation experiment. For M0- to M1-like macrophage differentiation, RAW 264.7 cells were treated with IFN-γ (20 ng/mL) and LPS (100 ng/mL) whereas for the M2-like macrophage transformation treatment was with IL-4 (20 ng/mL) and IL-13 (20 ng/ mL) for 48 hours. Before the cytokine treatment 5 × 10^5^ RAW 264.7 cells were seeded in a 6-well plate containing DMEM, 10% FBS media, and 1% pen/strep overnight.

### Migration assay.

Migration assays were analyzed in a 24-well Boyden chamber. We seeded 1 × 10^5^ tumor cells on the bottom chamber containing the complete media with 10% FBS whereas the 5 × 10^4^ RAW 264.7 cells on the top chamber were suspended in 100 μL serum-free media. After incubation at 37°C for 48 hours, cells were fixed and stained with 0.1% crystal violet. Random fields were quantified using ImageJ.

### RNA-Seq library preparation and sequencing.

Tumor tissue samples collected from the GEMMs, both *Myc* knockin and p53 driven, were used for RNA-Seq. RNA samples underwent quality control assessment using the RNA tape on Tapestation 4200 (Agilent) and were quantified with Qubit Fluorometer (Thermo Fisher Scientific). The RNA libraries were prepared and sequenced at the University of Houston Seq-N-Edit core per standard protocols. RNA libraries were prepared with QIAseq stranded total RNA library kit (QIAGEN) using 500 ng input RNA. mRNA was enriched with Oligo-dT probes attached to pure mRNA beads (QIAGEN). RNA was fragmented, reverse-transcribed into cDNA, and ligated with Illumina sequencing adaptors. The size selection for libraries was analyzed using the DNA 1,000 tape Tapestation 4200 (Agilent). The prepared libraries were pooled and sequenced using NextSeq 500 (Illumina), generating ~10 million 2 × 76 bp paired-end reads.

### RNA-Seq analysis.

Paired-end reads were trimmed using trimGalore software (https://github.com/FelixKrueger/TrimGalore; commit ID 4edff97), mapped using STAR (68) against the UCSC mm10 genome build, and quantified with featureCounts (69). Differential expression analysis was performed using DESeq2 R package 1.28.1 (70). The *P* values were adjusted with Benjamini and Hochberg’s approach for controlling the false discovery rate. Significantly differentiated genes between the comparisons were identified by applying the criteria of adjusted *P* value < 0.05 and fold-change exceeding 1.5 times. Pathway enrichment analysis was carried out using the GSEA (http://software.broadinstitute.org/gsea/index.jsp) software package; significance was achieved for adjusted *q* value < 0.25.

### Global proteomic analysis.

Global proteomic analyses were performed through the BCM Proteomics Core for frozen tumor samples isolated from the *Myc*-knockin and p53-driven GEMMs. Tumor tissues were crushed on a liquid nitrogen–cooled steel block with mechanical action. The homogenized tissues were then transferred to Eppendorf tubes and resuspended in 50 μL of ammonium bicarbonate + 1 mM CaCl_2_, snap-frozen in liquid nitrogen, and thawed at 42°C. This freeze/thaw step was repeated 3 times, and then the samples were boiled at 95°C for 2 minutes with vortexing at 20-second intervals and kept for proteolytic digestion. After isolation, protein concentrations were measured with the Bradford assay. A total of 50 μg of total protein was processed via 2-step trypsin digestion. First, proteins were digested with a 1:20 solution of 1 μg/μL trypsin/protein in ABC solution (50 mM ammonium bicarbonate, 1 mm CaCl_2_) overnight at 37°C with shaking. Next, additional digestion was carried out with a 1:100 solution of 1 μg/μL trypsin/protein for 4 hours in the same conditions. After the addition of 10% formic acid at 1:10 volume to neutralize the reaction, an equal volume of 80% acetonitrile + 0.1% formic acid was added to extract the peptides. Peptides were centrifuged at 10,000*g*, and the peptide concentration of the supernatant was measured using the Pierce Quantitative Colorimetric Peptide Assay (catalog 23275, Thermo Fisher Scientific). A total of 50 μg of the peptide was vacuum dried and stored at 4°C before resuspension for fractionation (if applicable) and sequencing.

### TARGET and R2 data set analysis.

The TARGET OS patient RNA-Seq data set (phs000468) was downloaded from dbGAP. Paired-end sequencing reads were trimmed using trimGalore, mapped using STAR alignment software against the human genome build UCSC hg38, and quantified with featureCounts. Differential expression analysis and GSEA were performed as described above under *RNA-Seq analysis*. Survival analysis was performed using patient clinical data plotted in GraphPad Prism (version 9.3.1). The secondary OS data set (GSE33382) was analyzed using the R2: Genomics Analysis and Visualization Platform (20).

### PDXs.

The OS PDXs analyzed in the study were acquired through our institutional protocol H-32668. The PDXs were previously reported (71).

### Statistics.

For all figures, Student’s 2-sample *t* test was used to determine if there was a statistical difference between the means of 2 groups (e.g., control and experimental groups). All *P* values were 2 sided, and a *P* value less than 0.05 was considered statistically significant. Quantified data shown represent at least 3 independent experiments. Data were represented as mean ± SEM. Log-rank (Mantel-Cox) tests were performed for the Kaplan-Meier analyses.

### Study approval.

The Baylor College of Medicine Animal Care and Use Committee approved the experimental protocol (AN-5225).

### Data availability.

RNA-Seq (original) data for this paper were deposited to the NCBI’s Gene Expression Omnibus under accession number GSE231821. Values for all data points in graphs can be found in the Supporting Data Values file.

## Author contributions

BKN contributed to coordinating and designing the study; developing, acquiring, analyzing, and interpreting the data; and drafting the manuscript. TDP contributed to the acquisition, analysis, and interpretation of the data and reviewed and edited the manuscript. LK and RS contributed to the acquisition and analysis of the data. AD, NR, and CC participated in reviewing and editing the manuscript. JTY participated in the coordination and design of the study, interpretation of data, and revision and review of the manuscript. All authors read and approved the final manuscript.

## Supplementary Material

Supplemental data

Supplemental table 1

Supporting data values

## Version 1 06/06/2023

In-Press Preview

## Version 2 07/10/2023

Electronic publication
