# Supplementary material for: MYC regulates CSF1 expression via microRNA 17/20a to modulate tumor-associated macrophages in osteosarcoma
Source: JCI Insight. 2023 Jul 10;8(13):e164947. doi: 10.1172/jci.insight.164947 (PMC10371352; doi:10.1172/jci.insight.164947)
Supplement: Supplemental data [file jciinsight-8-164947-s169.pdf]

A

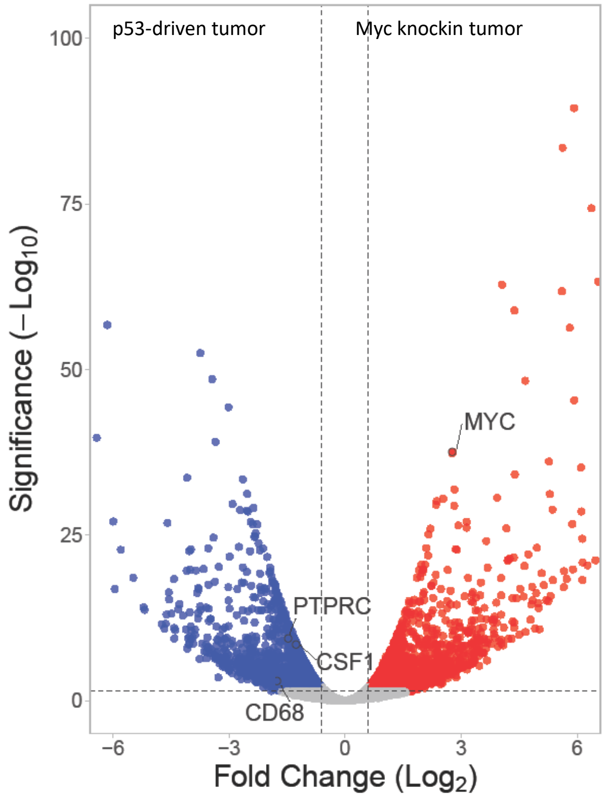

B

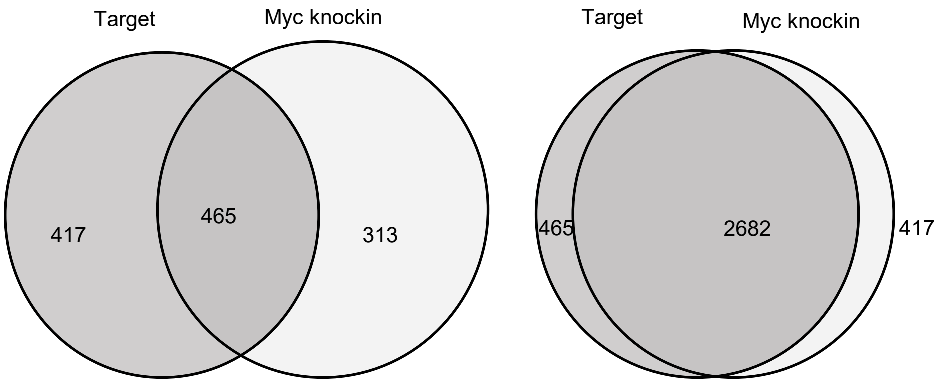

Supplemental Figure 1. Transcriptomic analysis of *Myc*-knockin GEMM and cross-species pathway analysis. **(A)** Volcano plot diagram showing the differential gene expression between Myc knockin (n=5) tumors compared to the p53-driven tumor (n=4) **(B)** Venn diagram showing the overlapping positively (left panel) and negatively enriched (right panel) pathways between the TARGET data OS patients' samples with high *MYC* expression and the *Myc* knockin GEMM samples.

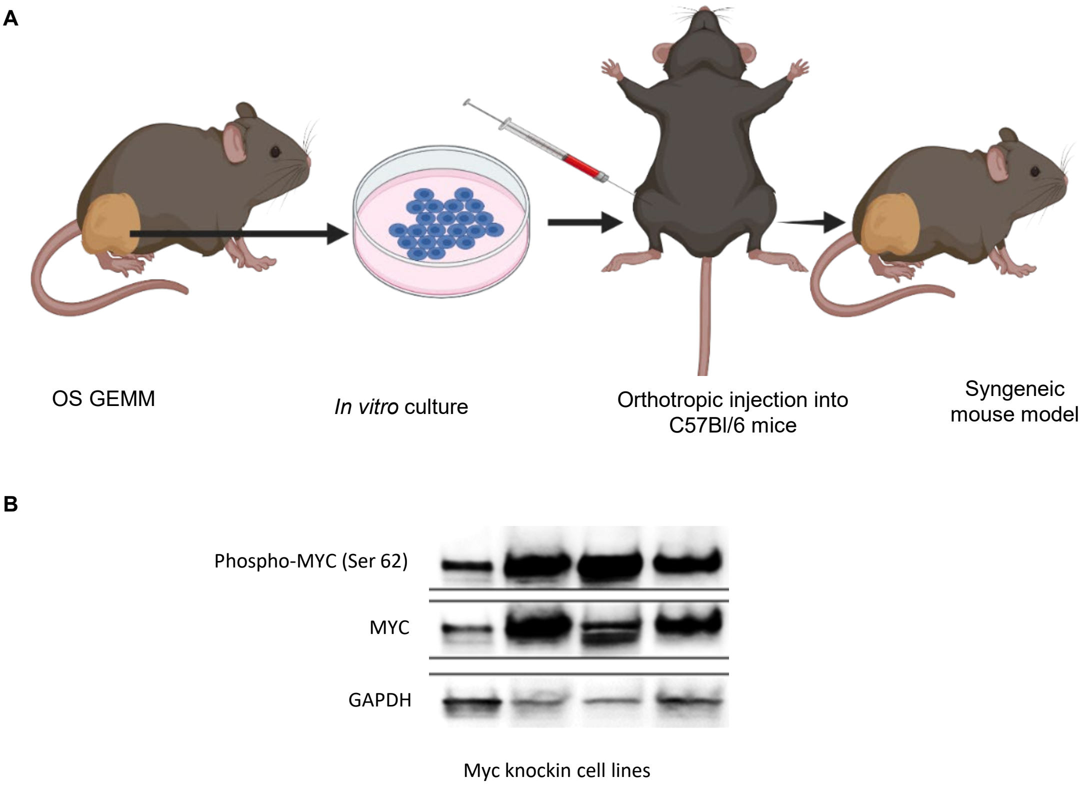

Supplemental Figure 2: (A) Schema of syngeneic mouse model and cell line generation. **(B)** Western blot demonstrating expression of MYC and p62-MYC protein expression in *Myc* knockin cell lines.

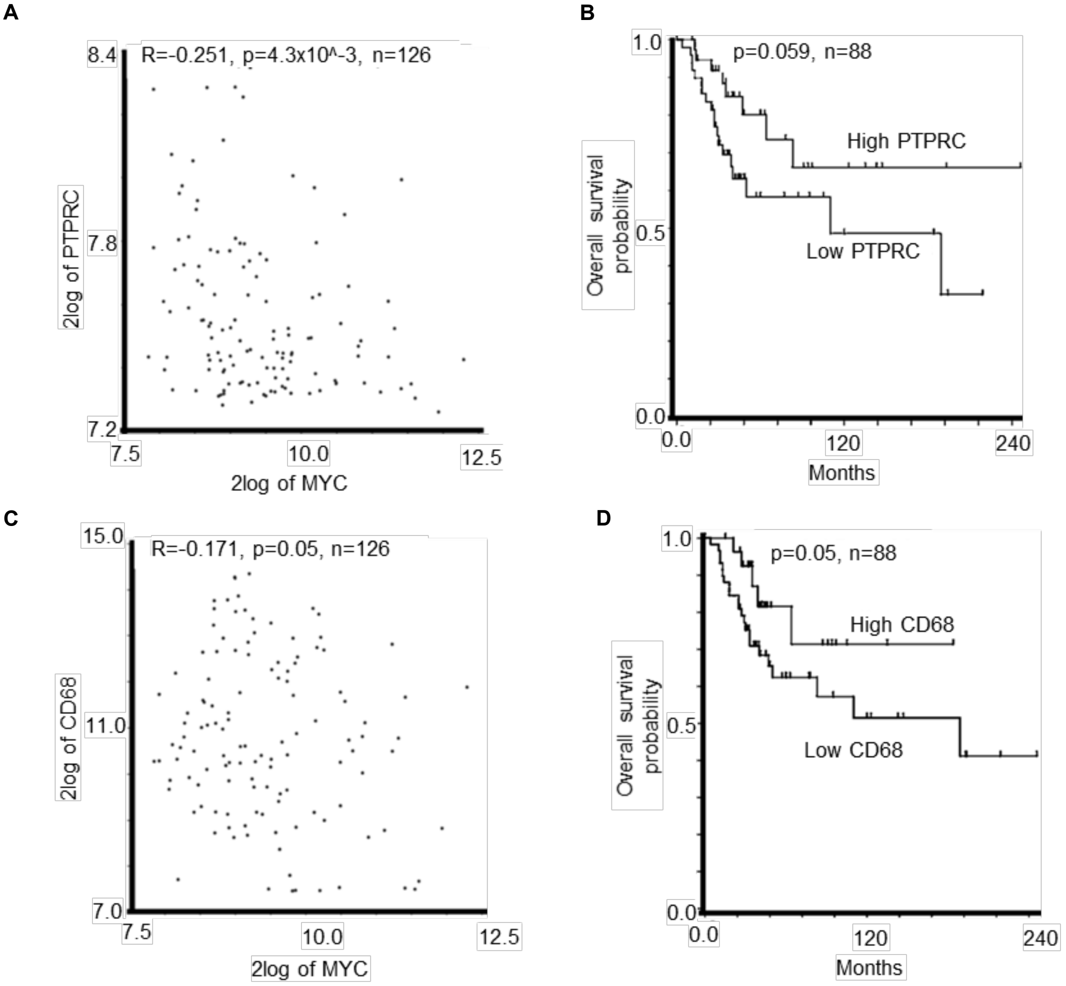

Supplemental Figure 3. R2 data set correlation between the *Myc* and *PTPRC/CD68* mRNA expression and their association with the overall survival. **(A)** Negative correlation between the *MYC* and *PTPRC* mRNA expression in human R2 dataset patients. **(B)** Kaplan-Meier curve of human R2 dataset for high and low *PTPRC* expression samples. **(C)** Negative correlation between the MYC and *CD68* mRNA expression in human R2 dataset patients. **(D)** Kaplan-Meier curve of human R2 dataset for high (blue) or low (red) *CD68* expression samples.

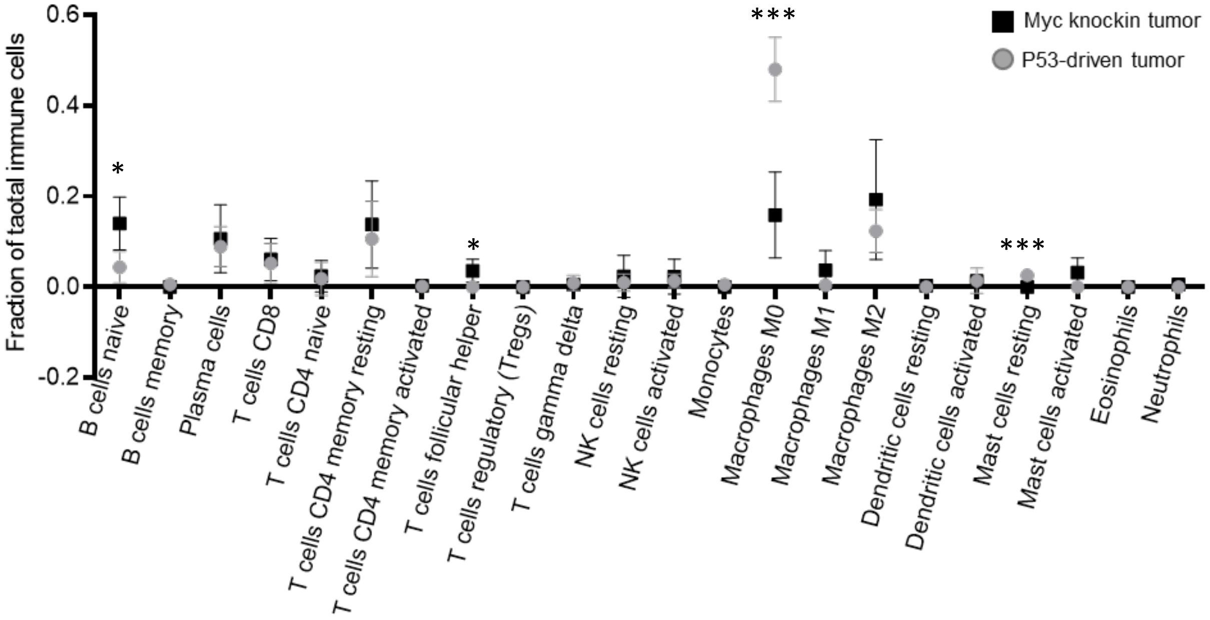

Supplemental Figure 4. CIBERSORT analysis of RNAseq data of *Myc* knockin and p53-driven tumor tissue samples. *p<0.05, ***p<0.001

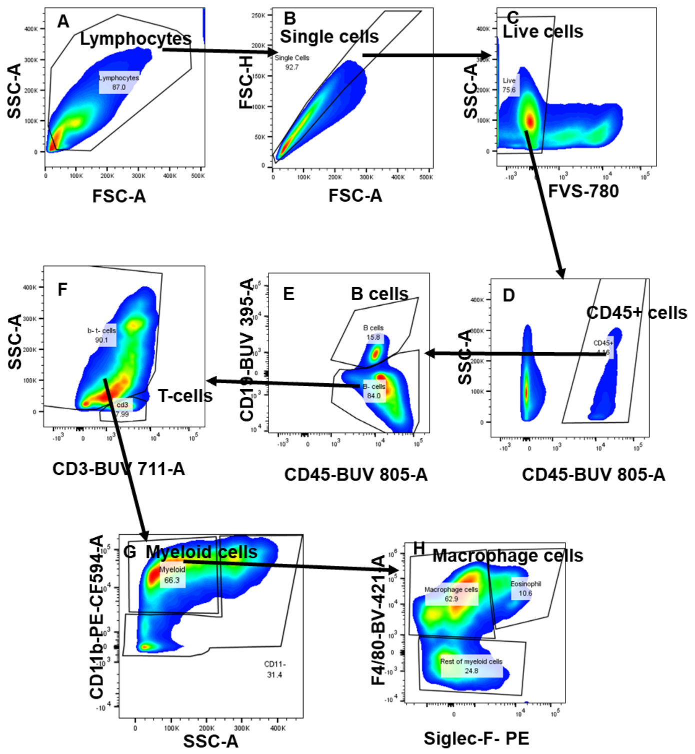

Supplemental Figure 5. Gating strategy for the FACSymphony analysis

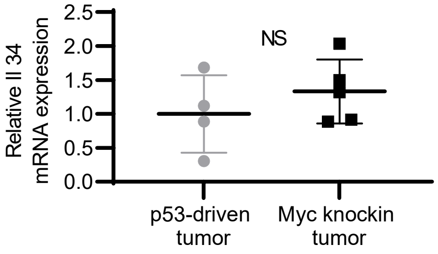

Supplemental Figure 6. Relative mRNA expression analyzed by the RNA sequencing demonstrated no significant difference in the Il34 mRNA expression in *Myc* knockin (n=5) tumors compared to the p53-driven (n=4) tumor sample.

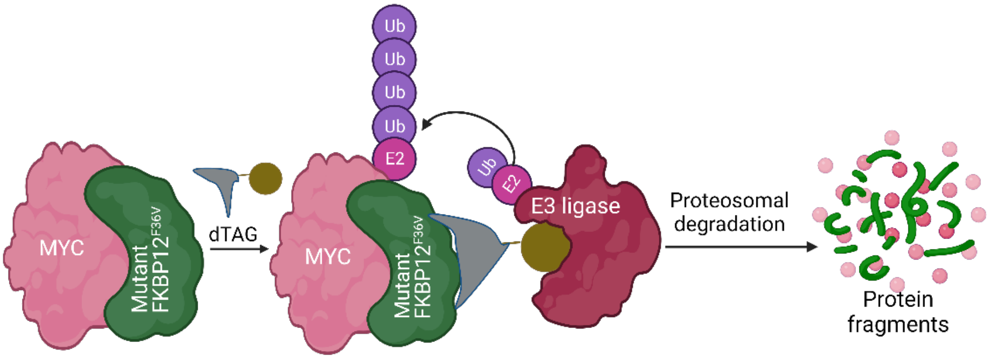

Supplemental Figure 7. Schematic diagram showing dTAG-MYC-protein degradation

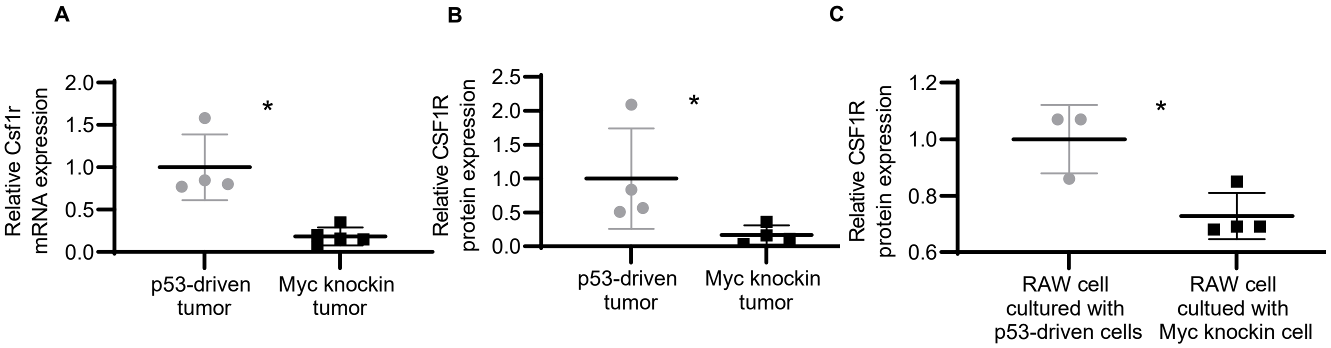

Supplemental Figure 8. Role of *Myc* in *Csf1r* regulation (**A**) Relative *Csf1r* mRNA expression analyzed by the RNA sequencing demonstrated lower MYC mRNA expression in *Myc* knockin (n=5) tumors compared to the p53-driven (n=4) tumor sample. **(B)** Protein expression analyzed by the total proteome analysis using mass spectroscopy demonstrating reduced CSF1R protein expression in *Myc* knockin tumors (n=4) compared to p53 -driven (n=4) tumor sample. **(C)** Relative mRNA expression of Csf1r in RAW 264.7 cells, cultured with *Myc* knockin (n=4) and p-53 driven cell lines (n=3). (*p<0.05).

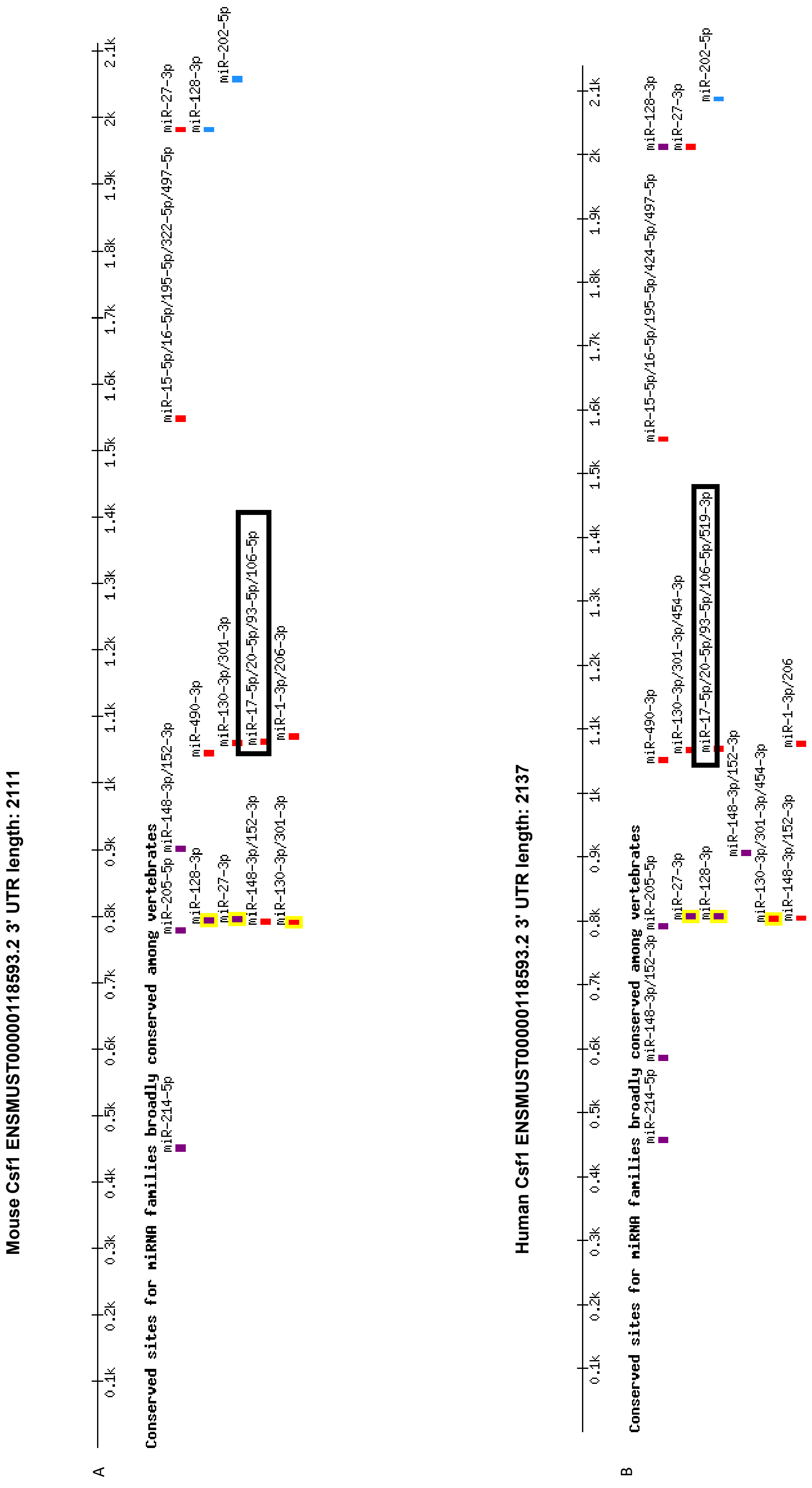

Supplemental Figure 9. (A) miR17-5p/20a binding region on the mouse CSF-1 UTR region. **(B)** miR17-5p/20a binding region on the human CSF-1 UTR region.

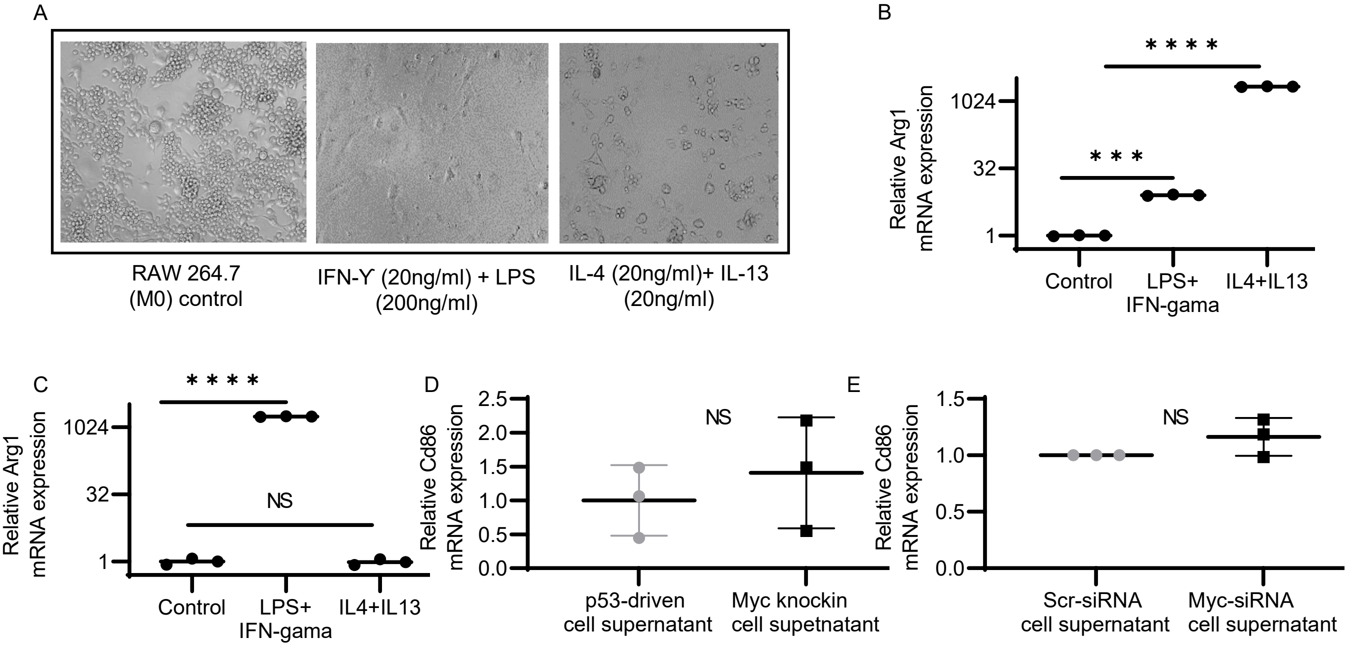

Supplemental Figure 10. Cell polarization after cytokines treatment. (A) RAW 264.7 cells were treated with either IFN-Ƴ (20ng/ml) + LPS (200ng/ml) or IL-4 (20ng/ml)+ IL-13 (20ng/ml) for 48 hours. The left image is an untreated control sample, the middle one is treated with IFN-Ƴ (20ng/ml) + LPS (200ng/ml) that showed M1-like macrophage morphology, and the right image shows the RAW cells treated with IL-4 (20ng/ml)+ IL-13 (20ng/ml) resembles with the M2-macrophage like morphology. (B) qPCR analysis for *Arg1* expression in RAW 264.7 cells after IFN-Ƴ (20ng/ml) + LPS (200ng/ml) or IL-4 (20ng/ml)+ IL-13 (20ng/ml) treatment. (C) qPCR analysis for *Cd86* expression in RAW 264.7 cells after IFN-Ƴ (20ng/ml) + LPS (200ng/ml) or IL-4 (20ng/ml)+ IL-13 (20ng/ml) treatment). (D) Expression of *Cd86* (M1-macrophage marker) in the RAW 264.7 cells cultured in the supernatant collected from the *Myc* knockin cell lines (n=3) as compared to p53-driven (n=3) samples. (E) Expression of *Cd86* in RAW 264.7 cells cultured in the conditioned media after *Myc-KD* using *siRNA* (n=3). (***p<0.001, ****p<0.0001).

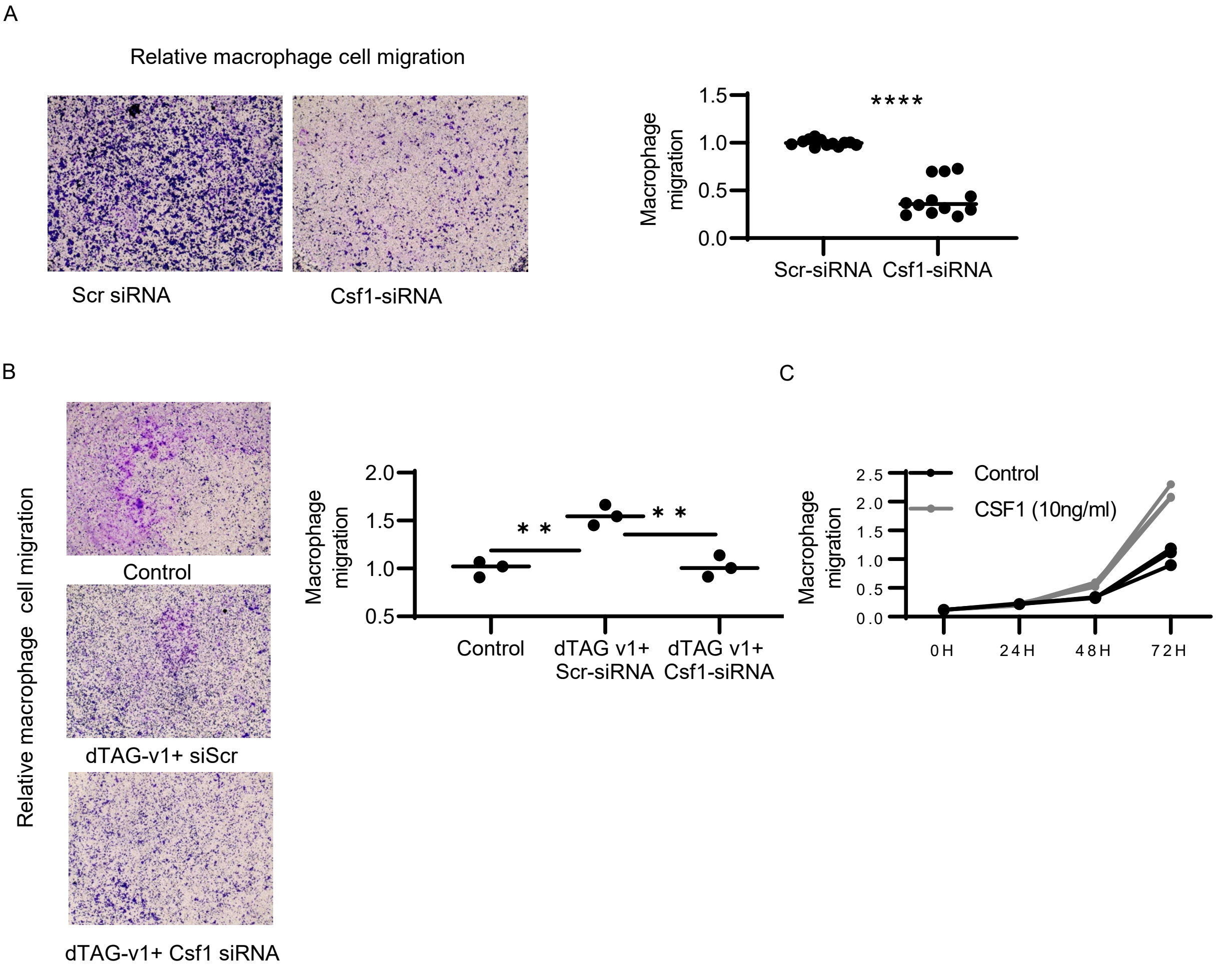

Supplemental Figure 11: Growth kinetics and migration of macrophage cells in the presence of Csf1 and MYC protein degradation. (A). RAW 264.7 cell migration after siCsf1 treatment compared to Scr control (B). RAW 264.7 cell migration after dTAG-v1 MYC protein degradation (middle panel) flowed by the siCsf1 treatment (right panel). (C) RAW 264.7 cells were treated with CSF-1 (10ng/ml) for 72 hours and the cell proliferation was analyzed using the CCK-8 assay. Macrophage cell proliferation after the CSF-1 treatment (light gray) compared to the control (Black line). (**p<0.01, ****p<0.0001).

Supplemental Table 2

Supplemental Table 2A. The list genotyping primer sequences

**Myc WT knockin**		
Rosa 1447R	GGAGCGGGAAATGGATATG	300
Rosa 884F	AAAGTCGCTCTGAGTTGTTAT	
**Myc T58A knockin**		
Myc655F	GACTCCGTACAGCCCTATTTC	1200
Ha-R	CTGGAACATCGTATGGGTACC	
**p53**		
Ld3F:	AAGGGGTATGAGGGACAAGG	LoxP-584
Ld4R:	GAAGACAGAAAAGGGGAGGG	Wt-431
**Cre**		
Cre1F:	TTACTGACCGTACACCAAAATTTGCCTGC	450
Cre1R	CCTGGCAGCGATCGCTATTTTCCATGAGTG	

Supplemental Table 2B. The list of PCR primer sequences

**S.N.**	**Target gene**	**Primer sequence**	
1	Murine Actb	F	5'-CGGTTCCGATGCCCTGAGGCTCTT-3'
		R	5'-CGTCACACTTCATGATGGAATTGA-3'
2	Murine Myc	F	5'-ATGCCCCTCAACGTGAACTTC-3'
		R	5'-GTCGCAGATGAAATAGGGCTG-3'
3	Murine Csf1	F	5'-GTGTCAGAACACTGTAGCCAC-3'
		R	5'-TCAAAGGCAATCTGGCATGAAG-3'
4	Murine Csf1r	F	5'-TGTCATCGAGCCTAGTGGC-3'
		R	5'-CGGGAGATTCAGGGTCCAAG-3'
5	Murine Cd86	F	5'-CATGGGCTTGGCAATCCTTA-3'
		R	5'-AAATGGGCACGGCAGATATG-3'
6	Murine Arg1	F	5'-GTGAAGAACCCACGGTCTGT-3'
		R	5'-CTGGTTGTCAGGGGAGTGTT-3'
7	Murine Rnu6	F	5'-CTCGCTTCGGCAGCACA-3'
		R	5'-AACGCTTCACGAATTTGCGT-3'
8	Human ACTB	F	5'-AGGCACCAGGGCGTGAT-3'
		R	5'-GCCCACATAGGAATCCTTCTGAC-3'
9	Human MYC	F	5'-GTCAAGAGGCGAACACACAAC-3'
		R	5'-TTGGACGGACAGGATGTATGC-3'
10	Human CSF-1	F	5'-TGGCGAGCAGGAGTATCAC-3'
		R	5'-AGGTCTCCATCTGACTGTCAAT-3'

F: forward primer, R: reverse primer
